# An in-silico study of cancer cell survival and spatial distribution within a 3D microenvironment

**DOI:** 10.1038/s41598-020-69862-7

**Published:** 2020-07-31

**Authors:** Marilisa Cortesi, Chiara Liverani, Laura Mercatali, Toni Ibrahim, Emanuele Giordano

**Affiliations:** 1 Department of Electrical, Electronic and Information Engineering “G. Marconi”, University of Bologna, Cesena, FC Italy; 2 Osteoncology and Rare Tumors Center, Istituto Scientifico Romagnolo Per Lo Studio E La Cura Dei Tumori (IRST) IRCCS, Meldola, FC Italy; 3 Advanced Research Center On Electronic Systems (ARCES), University of Bologna, Bologna, BO Italy; 4 BioEngLab, Health Science and Technology, Interdepartmental Center for Industrial Research (HST-CIRI), University of Bologna, Ozzano Emilia, BO Italy

**Keywords:** Computational models, Biomedical engineering

## Abstract

3D cell cultures are in-vitro models representing a significant improvement with respect to traditional monolayers. Their diffusion and applicability, however, are hampered by the complexity of 3D systems, that add new physical variables for experimental analyses. In order to account for these additional features and improve the study of 3D cultures, we here present SALSA (ScAffoLd SimulAtor), a general purpose computational tool that can simulate the behavior of a population of cells cultured in a 3D scaffold. This software allows for the complete customization of both the polymeric template structure and the cell population behavior and characteristics. In the following the technical description of SALSA will be presented, together with its validation and an example of how it could be used to optimize the experimental analysis of two breast cancer cell lines cultured in collagen scaffolds. This work contributes to the growing field of integrated in-silico/in-vitro analysis of biological systems, which have great potential for the study of complex cell population behaviours and could lead to improve and facilitate the effectiveness and diffusion of 3D cell culture models.

## Introduction

Cell culture is currently experiencing a fundamental shift from traditional 2D to 3D systems, that are more realistic representations of a biological tissue. These novel approaches, that integrate important aspects of cellular habitat, such as a non-uniform microenvironment, more complex diffusion processes and cell interactions with local physical features of the synthetic extracellular matrix (ECM), are bound to provide fundamental insights into cell biology, generating a closer approximation of reality as shown in^1–4^.

However, in order for 3D systems to become the standard in-vitro cell culturing technique, several issues still need to be addressed. In fact, the increased complexity in structural properties is, at the same time, the added value of these experimental systems and the limitation in optimising biological assays and protocols originally standardised for cells grown on 2D plastic surfaces. This complicates experimental design and data collection. While innovative 3D native assays are being developed^5,6^, computational models can complement the wet-lab experimental activity and help to address some of the limitations of 3D culture settings^7–9^.

The mathematical formalization of complex behaviours, however, is often maintained separate from the experimental analysis. As an example there are multiple models describing cancer-related cellular processes^10–17^ and the effect of antineoplastic therapies^18–24^ but most of them are presented as theoretical frameworks that do not aim at driving the experimental activity.

To integrate this important functionality we developed a general purpose scaffold simulator named SALSA that can be programmed to reproduce the behaviour of a population of arbitrary cells, grown in 3D scaffolds of tunable size and material. It relies on a custom hybrid continuous/discrete framework that is particularly beneficial for the representation of complex multicellular systems, and their interactions with the environment^25^ and grants more flexibility (highly customizable cell behaviour, interaction with the environment and resources utilization) than available agent-based simulators (e.g. NetLogo^26^, CompuCell3D^27^. SALSA combines a discrete model conceptually similar to Norton et al.^28^ and a continuous one akin to Cowan et al.^29^ to describe different aspects of the considered system. The former is used to model cells, their status and position within the scaffold, while the latter allows for the accurate simulation of continuous quantities (i.e. oxygen, glucose, Young’s modulus). This choice effectively realizes a simplified finite element / agent based combined model^30–32^ that allows for the representation of the main features of the in-vitro setup while limiting the computational cost.

Additionally SALSA was developed so as to incapsulate all the characteristics of the experimental model in configuration files separate from the simulator code. This choice minimizes the programming knowledge required to use it, thus making SALSA accessible to a larger user base than other available simulators (Chaste^33^, PhysiCell^34^, BioFVM^35^).

The most relevant feature of this system, however is its strong correlation with the experimental analysis. Indeed most of the parameters were inferred from in-vitro data and an extensive validation was conducted. As such, SALSA could be potentially used to pilot further experimental analyses, that could benefit from the possibility of testing in-silico a number of distinct configurations to identify the most suited for each application. Wet-lab results can conversely be used to improve the accuracy and reliability of the model. This integrated approach has been shown to be successful in a wide range of applications, as in^36–38^ . Moreover, opposite to most in-vitro assays, SALSA monitors individual virtual cells, allowing to study their distinct behaviours.

In the following a complete description of SALSA will be provided, together with its validation against in vitro experiments. For these assays two populations of human breast cancer cell lines (MCF7 and MDA-MB-231) that display different phenotypes, were grown in collagen scaffolds. Luminal subtype MCF7 cells are associated with slow growth and low motility while the basal-like MDA-MB-231 cells are connected with invasive disease and poor prognosis^39^.

SALSA was developed to keep track of both the cellular (i.e. position, status and behaviour) and the microenvironmental (i.e. distribution of glucose and oxygen, mechanical properties of the ECM) relevant features . This allows for a more comprehensive analysis of the system of interest and to infer variables difficult to access in-vitro, like the spatial distribution of the cells within the 3D environment, the associated local changes in glucose and oxygen concentrations and the interactions among the cells and the ECM. These variables provide a much more accurate description of the studied system and offer useful insights for understanding its functioning.

These potentialities will be here exploited to optimize in-silico the initial population cardinality, i.e. the ideal number of cells to seed in a scaffold to maintain a uniformly distributed population throughout the experiment.

## Results

### Validation of SALSA

The validation of SALSA consisted in comparing, over time, a) the cell density measured in-vitro to the results obtained with the in-silico simulation, and b) the ability of the virtual cells of inducing a stiffening of the ECM comparable to what observed experimentally. This latter comparison is particularly important for the considered experimental system, where the two compared cell lines were shown to behave differently^39,40^. MDA-MB-231, in particular, demonstrated to be able to increase the compressive Young’s modulus of the scaffold by reorganizing its fibers and increasing their density.

**Figure 1:**
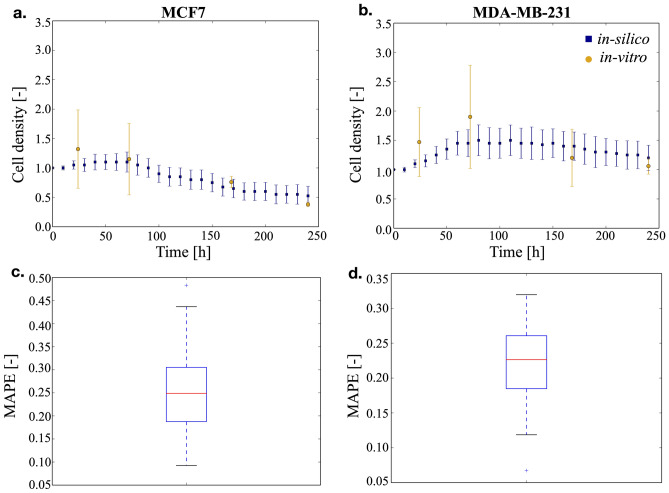
Time course of the cell density within the scaffold and concordance between in-silico and in-vitro data. In (**a**) MCF7 cells are considered, while in (**b**) MDA-MB-231 cells are shown. Both the experimental (circles) and the simulated (squares) data are reported as average and standard deviation. For graphical clarity only one point every 10 h is reported for the in-silico results. Panels (**c**) and (**d**) report the distribution of the mean average percentage error (MAPE) for MCF7 and MDA-MB-231 cells respectively.

As detailed in the methods section, the cell density was defined as the number of living cells with respect to the initial population and it was evaluated for the two different breast cancer cell lines over a period of 10 days. Every condition was simulated 50 times and the average results are shown in Fig. 1a,b. as square markers, while the data obtained in-vitro are shown as circles. A good agreement between the two datasets is observed throughout the whole considered time frame. Indeed, the distribution of the mean average percentage error (MAPE, Eq. 1, Fig. 1c,d.) was shown to have a median within the experimental variability (22% and 25% for the MDA-MB-231 and MCF7 cells respectively) despite an initial slightly larger distance among in-silico and in-vitro data, likely caused by variability in the efficiency of the in-vitro cell seeding procedure, that was not represented in the model.

1 $$ MAPE = \frac{{\left| {silico - vitro} \right|}}{vitro} $$

The differential behavior of the two cell lines is evident. Epithelial-like MCF7 cells, Fig. 1a, show an initial short proliferative period, followed by a rapid decrease in the number of cells that leads, at the end of the simulation, to a cellular density that is approximately half the initial one. On the contrary, mesenchymal-like MDA-MB-231 cells, in Fig. 1b, proliferate at a nearly constant rate for the first 3 days. Afterwards, the cell density decreases and reaches at day 10 a value comparable to the initial one. This limited proliferation has been observed in other 3D cell culture systems^41^, where it was associated with the dynamics of diffusion of nutrients and oxygen through the matrix.

**Figure 2:**
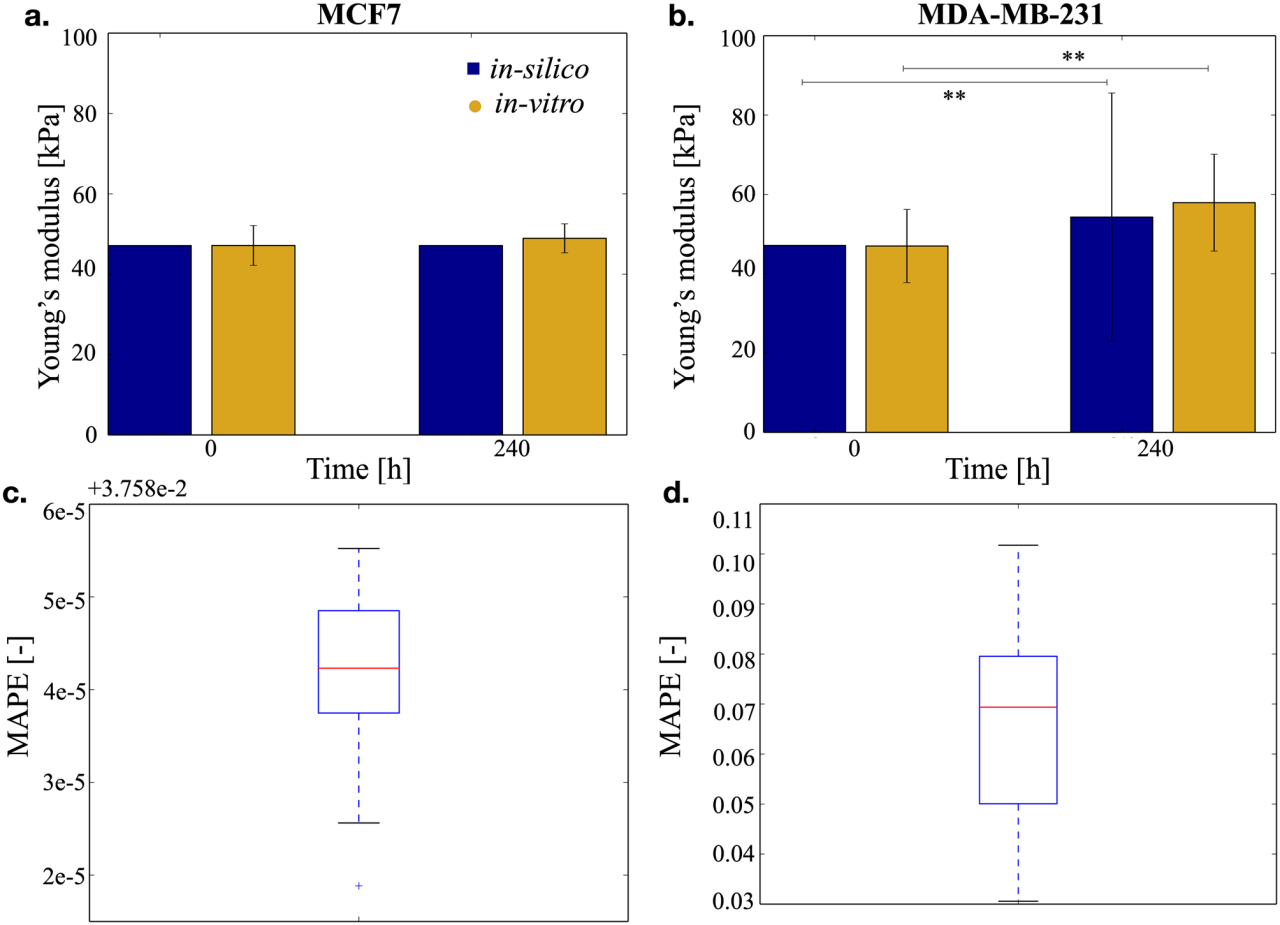
Effect of the two cell populations over scaffold’s stiffness. (**a**) MCF7 cells do not modify significantly their environment, thus the Young’s modulus is comparable when measured at day 0 and 10. (**b**) MDA-MB-231 cells, on the other hand, increase the stiffness of the scaffold. Data are reported as average and 95% confidence interval. In this case the error (**c** and **d**), computed as in 1 is much lower and coherent with the variability measured in-vitro.

The differential behavior of the two cell lines is also reflected in their ability of modifying their environment. The effect breast cancer cells on the stiffness of collagen scaffolds was previously studied^39^. Briefly we observed that MDA-MB-231 cells can significantly increase its value from 46.9 ± 5.3 to 57.9 ± 7.0 kPa, 10 days after cells were put in culture within the scaffolds (Kruskal–Wallis test, *p* = 10^−6^). Conversely, MCF-7 cells did not induce significant modifications of the scaffold stiffness. This was shown to correlate with the expression of Lysil-Oxydase (LOX), an enzyme responsible for collagen cross-linking that is 1,000-fold more expressed in MDA-MB-231 compared with MCF7 cells. Including in SALSA this differential LOX expression leads to the results reported in Fig. 2.

Simulated data are again able to consistently replicate these experimental results (Kruskal–Wallis test *p* = 10^−20^), the only difference being the variability obtained at day 10 for the scaffolds where MDA-MB-231 cells were cultured (Fig. 2b). This higher 95% confidence interval is caused by an inherent difference between the two datasets. Indeed in-vitro variability was obtained as the 95% confidence interval of the average stiffness of different scaffolds, while in-silico it was computed as the dispersion of the Young’s moduli distribution, obtained combining all the available data. Using the former approach to elaborate the in-silico data leads to a 15-fold reduction in variability that however does not account for inter-scaffold differences. As such the latter approach, can be considered to be more accurate, especially for MDA-MB-231 cells that exert a significant activity on their environment.

**Figure 3:**
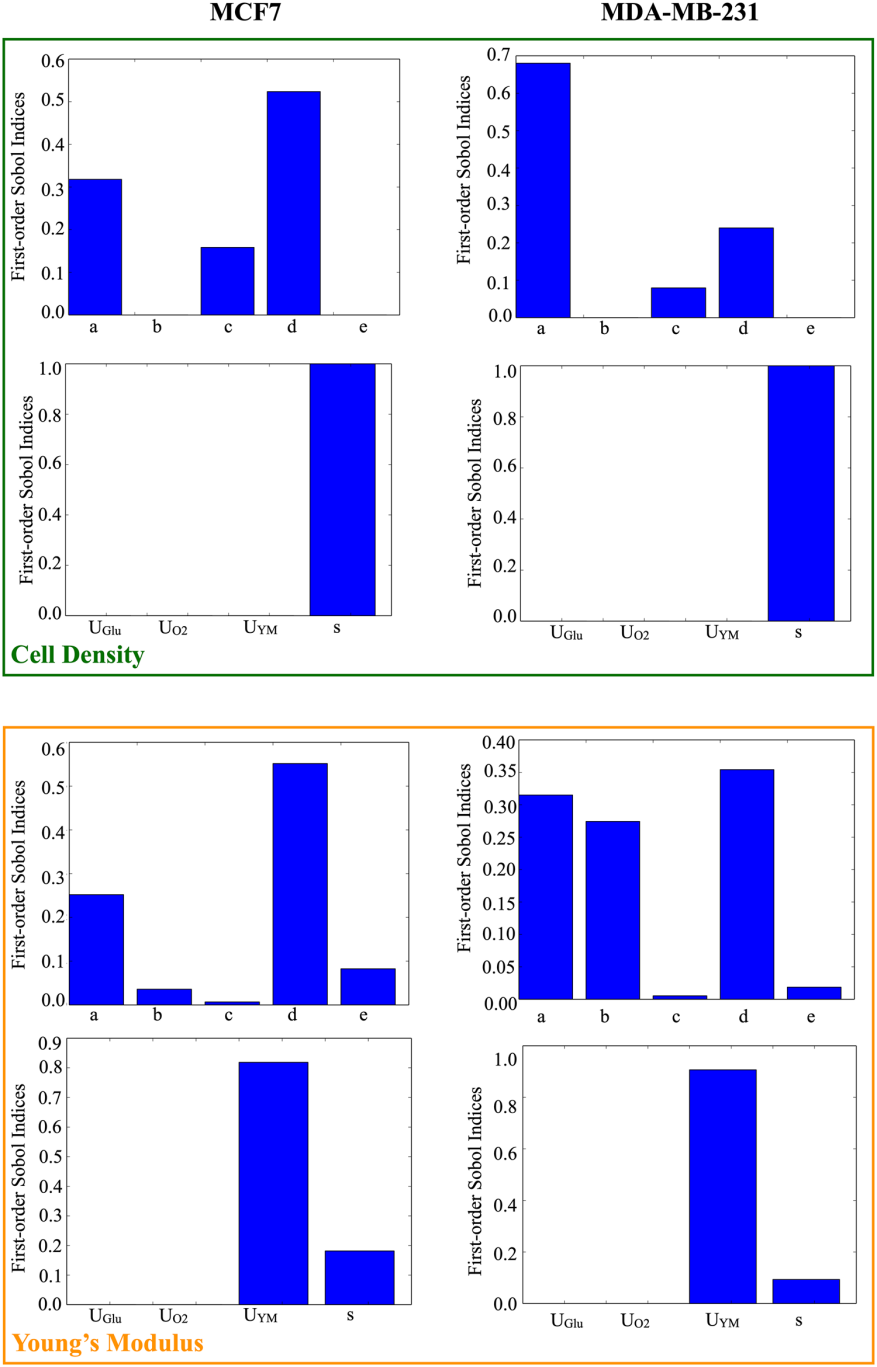
Analysis of the first order Sobol indices on the two considered outputs (cell density, Young’s modulus). Behavioural (**a**–**e**) and environmental (*U*_*Glu*_, *U*_*O*2_, *U*_*YM*_, s) parameters were analysed separately.

This analysis was completed by the study of how the main parameters of the model influence the results shown in Figs. 1 and 2. This was obtained through a global sensitivity analysis, fully detailed in the methods section, that allowed to compute the first order and total Sobol indices for each parameter (a, b, c, d, e, *U*_*Glu*_, *U*_*O*2_, *U*_*YM*_, s) listed in Table 1 (see also Fig. 3 and Supplementary Figs. 6, 7 and 8) and the associated temporal evolution of the standard deviation (Supplementary Fig. 9). First order and total Sobol indices were shown to be approximately equal showing that the model’s parameters have minimal interactions. The most relevant change pertains the behavioural parameter c, that drives the transition between proliferation and quiescency and its role in the determination of the Young’s Modulus. This is likely determined by the central role of proliferant cells in the modification of the surrogate matrix properties.

**Table 1:** Parameters considered for the global sensitivity analysis. See the supplementary material for a more extensive explanation.

Parameter	Description
a	Multiplicative coefficient of the doubling rate of the proliferant cells and of the degradation of the dead cells
b	Multiplicative coefficient of the rate of transition between quiescency and proliferation
c	Multiplicative coefficient of the rate of cell death
d	Multiplicative coefficient of the rate of transition between proliferation and quiescency
e	Multiplicative coefficient of the migration rate
U_Glu_	Amount of glucose consumed by each cell in the time unit
U_O2_	Amount of oxygen consumed by each cell in the time unit
U_YM_	Effect exerted by each cell on the matrix in the time uni
s	Scale factor between the oxygen and glucose consumed by quiescent and proliferant cells

Limiting the analysis to the first order indices, cell density was shown to be mainly determined by a, c, d, and s, albeit with different intensities, while the matrix stiffness was shown to be more influenced by a, b, d, e, *U*_*YM*_ and s. As described in the supplementary material, some of these parameters were determined from experimental data (a, e, s, *UY M)* while others were computationally optimized (b, c, d). This compromise was necessary due to the lack of experimental evidences regarding certain aspects of the model, like the transitions between different cell states. We assume that overfitting risk is minimized having extensively used literature data for parameter estimation. Moreover only part of the experimental data was used for this purpose. The resulting model was indeed able to predict the value of the rest of the experimental results.

Stochasticity, on the other hand, was shown (Supplementary Fig. 9) to progressively increase as the simulation proceeds. This is partly due to the uniform starting condition imposed in our simulations, and is manly determined by the probabilistic nature of the rules and the procedures used to update cell status. Environmental parameters were shown to dominate behavioural ones when considering the Young’s modulus as output, while both classes of parameters equivalently determined cell density.

In the following sections, we will present the use of this computational tool to study important aspects of 3D cultures that are difficult to assess experimentally, such as the relationship between cell localization and viability, the local matrix stiffness and the distribution of oxygen and glucose within the scaffold. Finally an example of how SALSA could drive the in-vitro analysis will be shown. The simulation of three alternative initial cell densities will be presented and the experimental condition capable of granting sustained growth of the virtual population will be identified.

### Study of local variables using SALSA

A fundamental characteristic of 3D cultures, that makes them more physiologically representative than their 2D counterpart, is that distinct locations within the scaffold display differential microenvironments due to the presence of a nutrients gradient from the external layer to the inner scaffold core. Measuring these differences in-vitro, however, is particularly challenging due to the lack of high resolution quantitative techniques.

**Figure 4:**
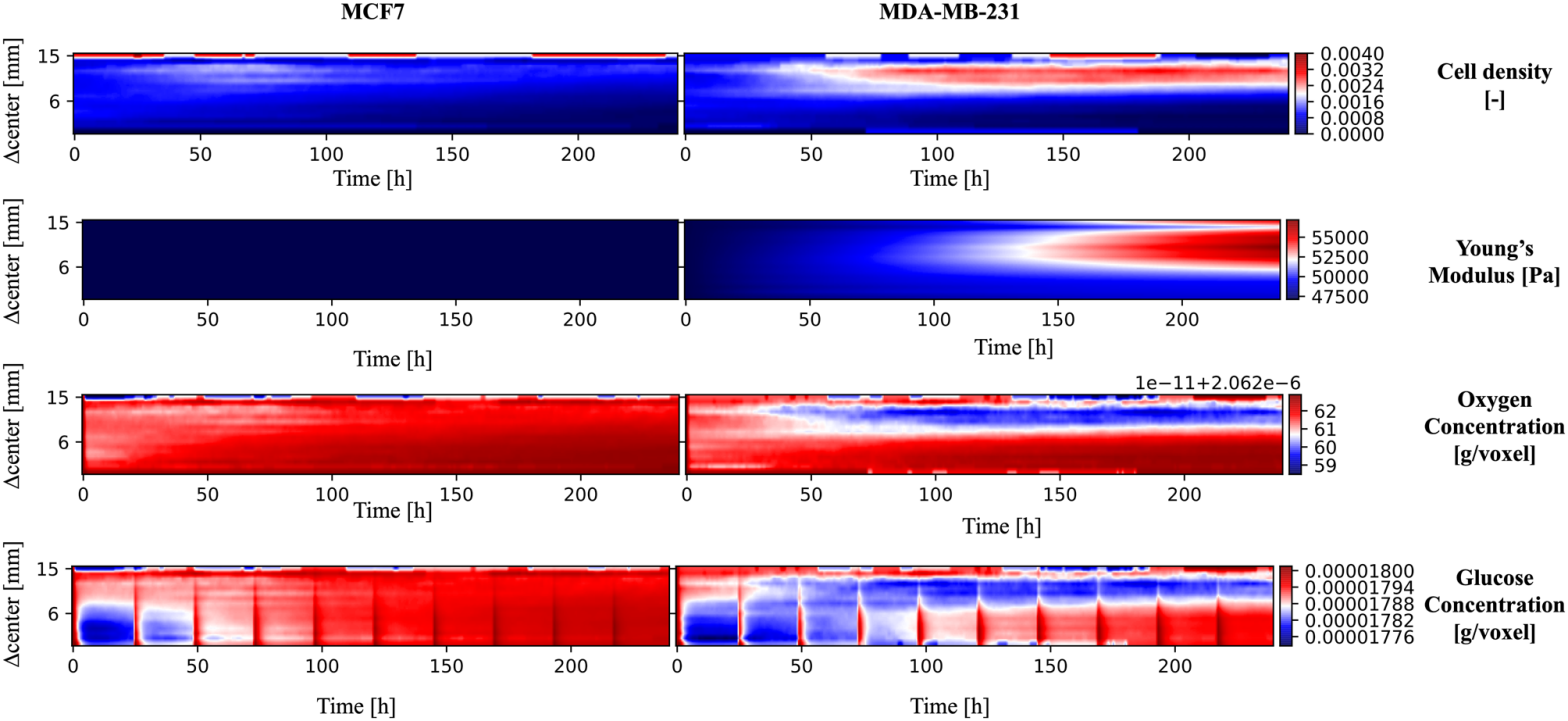
Evaluation of the influence of the distance from the scaffold center on the simulated variables. The color scale in the heatmaps represents the fraction of living cells normalized with respect to the cardinality of the initial population (cell density) or the average value of a specific variable (oxygen and glucose concentrations, Young’s modulus). A bilinear interpolation has been applied. The red vertical bands visible in the glucose concentration panels correspond to media changes and thus to the replenishing of glucose to its original concentration in the cell culture media.

SALSA can be used to address these limitations as it tracks the location of each cell and the distributions of oxygen, glucose and Young’s modulus with spatial and temporal resolutions of 1 mm and 1 h respectively. This information can be used to complement the experimental analysis and retrieve valuable information difficult to obtain otherwise. This concept is exemplified in Fig. 4, where the results of these simulations are represented highlighting the effect of the distance from the center of the scaffold on each variable. For the simulated results the Manhattan distance was substituted to the euclidean one, since the cubic lattice used for the simulation is not radially symmetric.

When considering cell density, the color scale represents the fraction of living cells normalized with respect to the cardinality of the initial population. Although relatively uniform at the beginning of the simulation, this value rapidly decreases in the scaffold core (∆ center < 6 mm) while it stabilizes (MCF7) or increases (MDA-MB-231) in the most peripherical regions. Albeit coherent with the global results presented in Fig. 1 this prediction points at the existence of two radically different micro-environments within the 3D structure, one compatible with cell growth and survival and another associated with population collapse. Imaging of a scaffold section with a confocal microscope confirmed this result in-vitro^40^. Indeed the more aggressive MDA-MB-231 cells were capable of migrating toward more favourable environments for survival, while MCF7 cells maintained their original approximately uniform distribution and were unable to proliferate effectively.

The high mortality rate in the scaffold core seems to be connected with glucose availability, as the initial (24–48 h) decrease in the average concentration of this nutrient in the core of the scaffold is synchronized with cell density reduction (Fig. 4). Notably, despite a comparable reduction in glucose level, MDA-MB-231 cells in the external layers of the scaffold are predicted to be in the proliferative status. Their proximity to nutrient and oxygen rich medium is a plausible explanation for this predicted behaviour.

Simulated oxygen levels don’t decrease prominently at the beginning of the simulation. Their lowest concentrations were registered for the MDA-MB-231 cell line toward the end of the experiment. This might be connected with both the higher diffusivity (more than fourfold) and the lower cell uptake (∼ 10^4^) of this molecule, when compared to glucose. The consequential reduced abruptness in the drop of oxygen concentration might reduce the impact of this variable on cell behaviour.

Finally the average simulated Young’s modulus displays two different behaviours for MCF7 and MDA-MB-231 cells. In the former there is no clear difference with respect to the initial condition, while the latter exhibit a dependence on the distance from the center of the scaffold similar to cell density distribution. Indeed a 10% difference in stiffness between the more rigid external shell and the softer core is predicted for MDA-MB-231 at the end of the simulation. This result suggests that these cells might generate an anisotropic material with more complex mechanical properties and behaviour than the initial structure.

The increased resolution granted by SALSA simulations was here shown to be potentially instrumental for the study of the complex feedback loops that govern the interaction between the cells and their environment, as the simplified dynamics and the immediate access to all the variables of interest could aid the optimization of the experimental setting and the interpretation of population-level results.

### Study of impact of differential initial population cardinality

**Figure 5:**
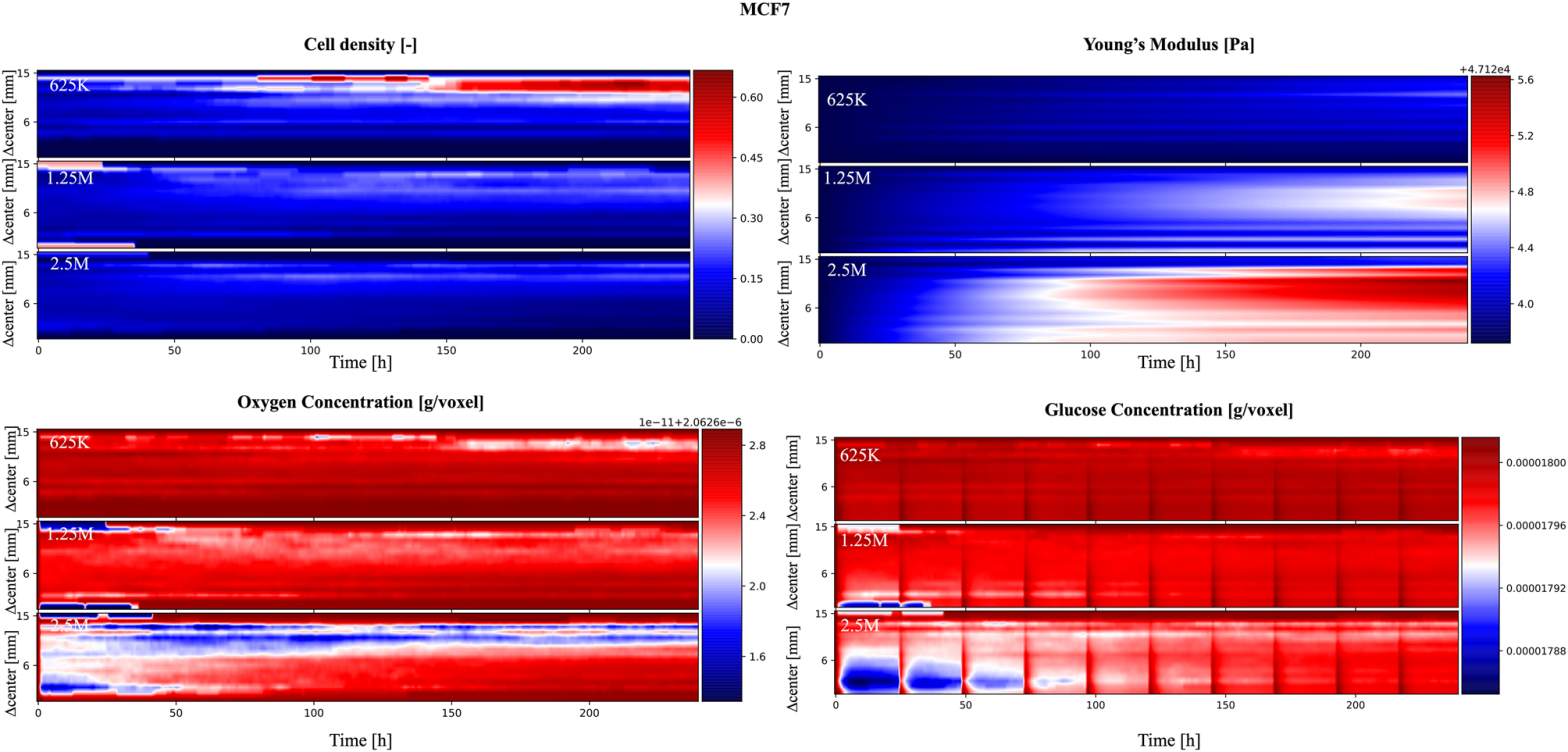
MCF7 cells. Analysis of the role of initial population cardinality on the simulated variables as a function of the distance from the scaffold center. The color scale are defined as in Fig. 4.

**Figure 6:**
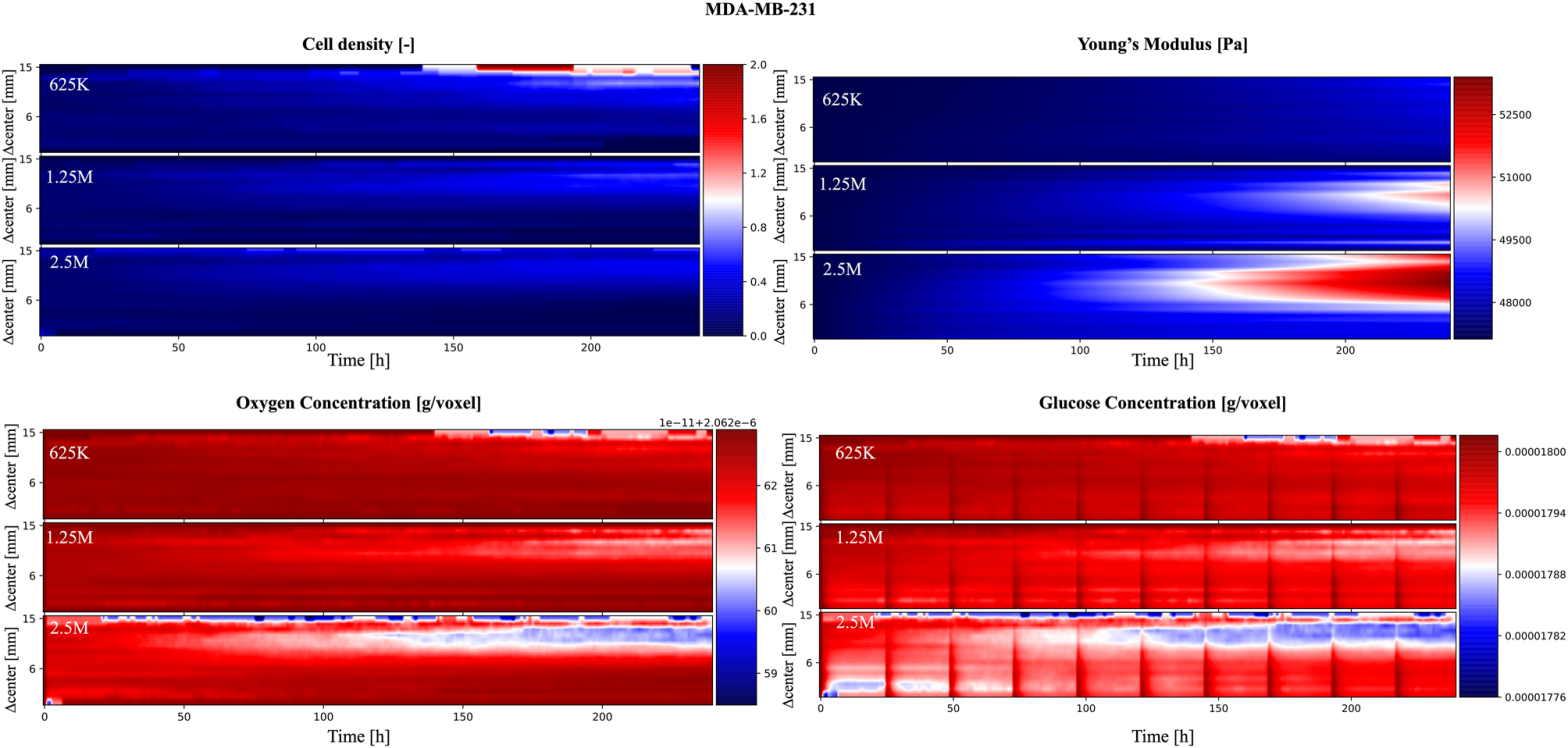
MDA-MB-231 cells. Analysis of the role of initial population cardinality on the simulated variables as a function of the distance from the scaffold center. The color scale are defined as in Fig. 4.

The analysis of the SALSA simulations described in the previous section highlights how an initial density of 5 M cells might not be ideal, as it was associated with non-viable conditions in the innermost part of the virtual scaffold. To test if a different initial population density could increase the uniformity of cell distribution and stiffness we simulated the same experiment considering starting populations of either 625 K, 1.25 M or 2.5 M cells. The results of this in-silico analysis are reported in Figs. 5 and 6, for MCF7 and MDA-MB-231 cell lines respectively.

In these simulations, a lower initial cell density importantly reduced the glucose depletion in the core region at the beginning of the experiment, especially when considering starting populations of 625 K and 1.25 M cells. This was associated with a more pronounced and uniform increase in cell density. A similar distribution was also observed in the simulated scaffold stiffness, where starting populations with fewer than 1.25 M cells were shown to be associated with a low intrascaffold variability (5% at most).

These predicted features are valuable drivers for the design of in-vitro 3D culture protocols, where more homogeneous cell populations are expected to reduce the inter-experimental variability and to increase the significance of outcome. To confirm these results, we used confocal microscopy for the imaging of the cell spatial distribution within the whole scaffold.

The initial seeding of 5 M MDA-MB-231 cells determined an edge region significantly more populated than the core, after 7 days of culture^40^, while an initial concentration of 1 M cells was associated with a more homogeneous cell distribution within the scaffold without statistically-significant differences observed over time between core and edge regions (Supplementary Fig. 10).

**Figure 7:**
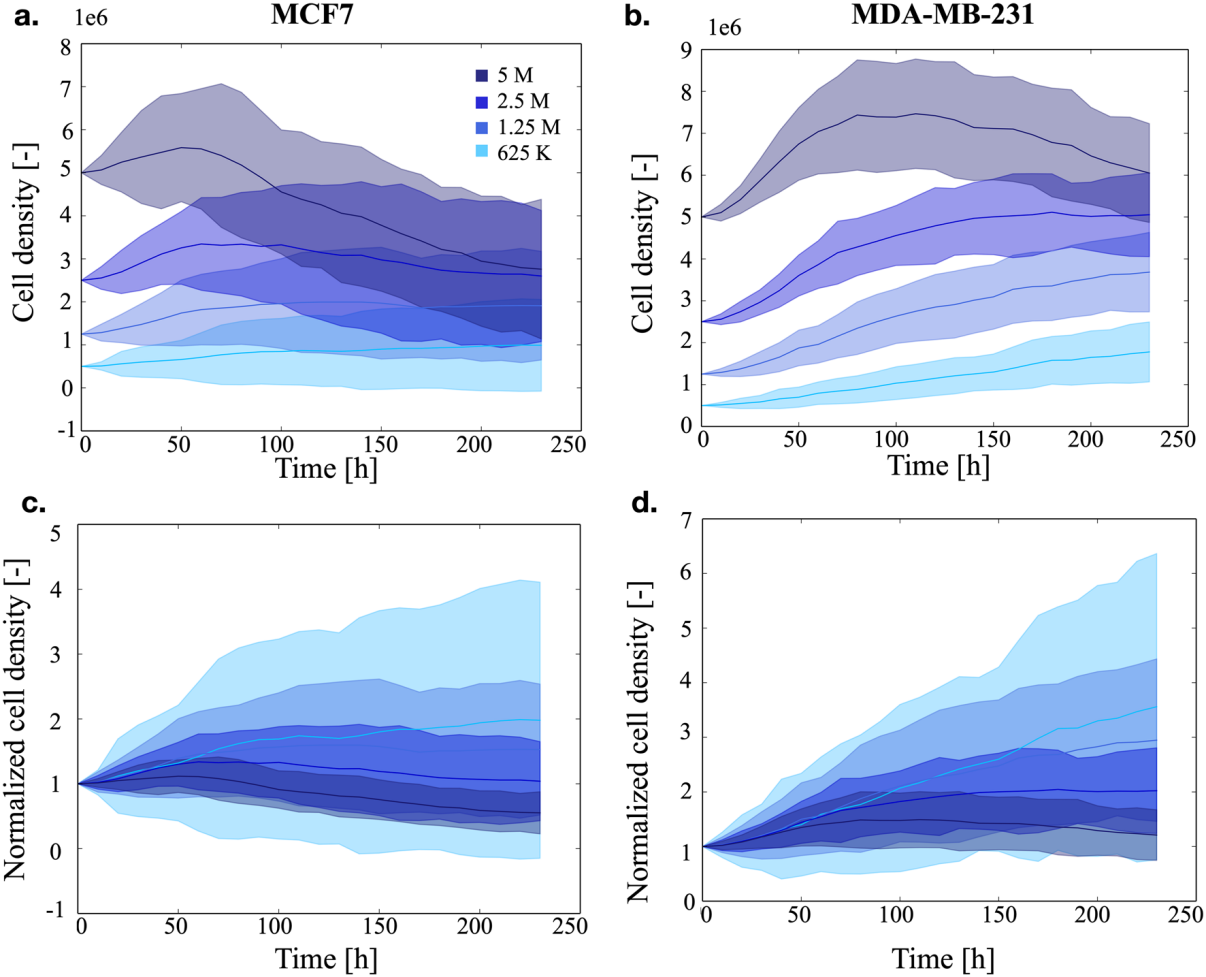
Analysis of the influence of the initial population cardinality on the number of live cells in the scaffold. In (**a**) the average number of cells (and 95% confidence intervals) obtained for the MCF7 cell line is shown, while (**b**) reports the same data for the MDA-MB-231 population. In panels (**c**) and (**d**) the same data in (**a**) and (**b**), normalized with respect to the initial population are plotted.

Additionally, a further analysis of our simulated data revealed that smaller initial cell populations, although being unable to generate the cell densities obtained seeding 5 M cells, achieved final cardinalities that were almost independent from the initial condition (Fig. 7a,b), that is coherent with the experimental results shown in Bitar et al. (2008)^42^. Room availability within the scaffold appears not to be the reason for this result, that is likely to be rather determined by the establishment of an equilibrium between cellular growth and nutrients availability.

Normalizing with respect to the initial population (Fig. 7c,d) highlights how lower population densities are associated with a more pronounced growth and generate populations that can be sustained by the system for the entire experiment.

The overall results of this section, while mainly qualitative and limited to cell density, show that an initial population of 625 K cells could be the best tradeoff between the amount of living cells and a homogeneous distribution within the scaffold at the end of the experiment. This is also supported by other experimental works^43–45^ that set the initial population cardinality between 300 K and 1 M cells.

## Discussion

In this paper we have presented SALSA, a general purpose computational tool that can be used to simulate significant features (e.g. proliferation and survival) of cell populations growing in 3D polymeric scaffolds, together with ECM inherent stiffness. The integrated discrete/continuous cellular automaton formalism chosen for this computational tool represents an effective compromise between accuracy and computational cost. Indeed, it corresponds to a simplified version of an agent-based/ finite element combined model^30–32^ that, having a lower spatial resolution, allows for the effective representation of the entire scaffold. This is particularly important as SALSA was developed to fully integrate with the experimental analysis that often results in global measurements across the whole 3D culture. An important limitation of this approach, however, is the need to reduce the size of the simulated population by a scale factor, computed to maintain the same initial relative occupancy of the scaffold (see methods section). This results in the aggregation of multiple biological cells in a single entity.

Within this work, we have shown how the proposed method makes it possible to study how factors like the distance from the center of the scaffold affect macroscopic properties like cell viability. This concept is exemplified in Fig. 4 where the analysis of 100 simulations identified the external layer of the scaffold as the most favourable environment for cell survival. Additionally the reduced availability of nutrients and oxygen, due to cell consumption together with the finite diffusion velocity of these molecules, were predicted being likely responsible for the uneven final cell distribution. Simulated data also point at glucose as a more critical environmental variable in determining scaffold core cell depletion.

SALSA can also be used to infer the behaviour of the 3D cell culture in untested conditions. This feature was here exploited to evaluate the effect of different initial population cardinalities on the dynamic evolution of cell density, with the aim of improving scaffold occupancy and reducing cell death (Fig. 7).

Another important characteristic of this model is the implicit integration of local interactions. Since this interplay between cells and ECM has a fundamental role in cell biology^46–48^, its intrinsic formalisation in cellular automata greatly improves the usefulness of the model and its ability of effectively reproduce the behaviour of biological systems of interest. This aspect is here exploited to represent the progressive scaffold stiffening caused by cell activity (Figs. 2, 4, 5, 6). Indeed this process was modelled as a local operation making proliferant cells capable of altering the Young’s modulus of their entire neighbourhood and thus affect the behaviour of other cells in their microenvironment.

The direct integration of in-silico and in-vitro results is another fundamental advantage of using cellular automata and their accurate representation of spatial environment. Indeed while theoretically less sound than other approaches (i.e. partial differential equations), the results obtained with this method are readily interpretable and could have a direct repercussion on the experimental analysis. Specific outputs of SALSA were shown (Figs. 1, 2) to be coherent with wet-lab generated results, opening to the potential of using this software to complement, and possibly substitute, the experimental study. As an example, the opportunity to efficiently carry out multiple simulations in a short time allows to screen a wide range of possible configurations of an experimental protocol, helping to define elements such as the time-points at which to carry out specific assays and/or the environmental conditions better suited for the biological process to investigate.

This strategy could also be used to identify the most interesting hypotheses to be tested in the lab. This integrated in-silico and in-vitro analysis is expected to bring significant advantages both in term of resources optimization (time and costs) and scientific impact, since the more efficient planning is likely going to lead to meaningful results in a shorter time.

Beside being able to replicate experimental data, SALSA appears able to infer, with high temporal (1 h) and spatial (1 mm) resolution, variables not readily available experimentally. In particular, how nutrients and oxygen availability affects cells according to their position within the scaffold could be predicted (Figs. 4, 5, 6) without the need of time consuming in-vitro analyses. These results were fundamental for hypothesizing the cause of cell death in the initial experiment and identify which aspect of the experimental protocol could be changed to address this issue. This feature sets SALSA apart from other relevant hybrid models in cancer research^15,22^ that mostly rely on theoretical models that are more difficult to incorporate with experimental analysis.

Lowering the initial cell density effectively reduced inter-scaffold variability although the central layers are still less vital, when compared to the external ones. The use of a perfusion bioreactor could potentially offset this difference, as dynamic culture conditions could increase oxygen and glucose levels within the scaffold, through active diffusion creating more favourable conditions and potentially allowing for cell growth in the inner layers. Within this context SALSA could be used to optimise flux velocity and initial population cardinality according to the experimental needs.

Additionally the high resolution of this software, that gives access to the distribution of several variables within the scaffold (e.g. cell status, nutrients and oxygen concentrations, Young’s Modulus) provides with the opportunity of quantifying phenotypic variability with higher accuracy, with respect to widely used in-vitro techniques. These generally evaluate only population averages and how these values change among different populations. This approach underestimates population diversity, as higher intra- versus inter-scaffold variability has to be expected. This concept is exemplified in Fig. 2, where the standard deviation computed from the simulated data is 15-fold higher than the experimentally measured value. Increasing the spatial resolution of in-vitro experiments, however, is technically very challenging, as it often requires the use of complex microscopy setups and the destruction of the sample (e.g. staining on paraffin-embedded slices). Computational approaches, such as the one here presented, could overcome these limitations, coupling standard in-vitro assays with an in-silico analysis capable of providing a more complete and exhaustive description of the system.

The results here presented show how combining experimental analysis and computational simulations could improve the study of complex 3D culture systems, providing scientists with more complete information and with tools for experimental conditions screening and optimization. Being entirely programmable, SALSA is a particularly innovative contribution, that could promote the diffusion and application of this integrated approach to the study of complex biological systems and could be determinant for the establishment of 3D culture systems as a standard, superseding traditional monolayer models. To this aim SALSA is released as a freeware software (https://www.mcbeng.it/en/category/software.html) and is amenable to several improvements.

Among them a non-random seeding mode would support the simulation of other 3D cell culture models (e.g. multicellular aggregates), while specific cell–cell communication mechanisms (i.e. direct interaction among neighbours, soluble factors) and cell–environment interactions (e.g. ECM degradation/remodeling, response to mechanical stresses) would increase the accuracy and versatility of this tool. The most relevant upgrade, however, would be the possibility of using SALSA to simulate pharmacological treatments.

This aspect could be implemented with minimal modifications, as the continuous variables modeling already in place could be exploited to simulate the dynamic evolution of drug concentrations and standard pharmacokinetic/pharmacodynamic data could inform the parameter selection procedure. This framework well adapts to a number of anti-cancer treatments (e.g. chemotherapy, target therapy, combination therapy) that rely on soluble factors. Additionally an *ad-hoc* function could be added to solve the linear Boltzmann transport equations and thus introduce the possibility of simulating radiotherapy.

As SALSA is particularly suited for the in-silico optimization of the experimental conditions, this new feature has important potential applications both for the development of new pharmacological agents, or their repurposing, and for the prediction of patient response. These are key aspects of in-silico medicine, a new discipline that aims at improving treatment effectiveness and optimizing the drug development process through the extensive application of computational models. The framework here described has several advantages in this regard, being freely available, programmable and seamlessly integrable with in-vitro analysis.

## Methods

### SALSA

SALSA is a computational tool for the simulation of 3D cell cultures in polymeric scaffolds. It uses a hybrid discrete/continuous cellular automaton to accurately describe the system, while containing the complexity and the computational cost. A 3D cubic lattice represents the main structure of the model and its size and resolution can be adjusted to fit the specific application (see the supplementary material for a sensitivity analysis of these parameters). This matrix is filled with integer values to represent the cells, their status and position within the scaffold, while real numbers describe the scaffold’s Young’s modulus and the distributions of glucose and oxygen. This simplified agent-based/finite element model can be programmed to describe different types of cells (e.g. dead, quiescent proliferant) and their interactions with the environment. SALSA rules are the main tool to model these aspects. They follow the syntax in Eq. (2) where *ID*_*rule*_ is an index univocally identifying each rule and brackets delimitate optional terms.

2 $$ ID_{rule} = A \to B\left( { + C} \right),D $$

SALSA rules can be divided in behavioural and environmental. While they both follow the same structural pattern (Eq. (2)), the former describe changes in cell status and macroscopic behaviours (e.g. duplication, migration, cell death) while the latter model the interaction with the environment. These differences provide alternative meanings for the A, B, C and D terms, as detailed in Table 2.

**Table 2:** Definition of each term in Eq. (2) for both the behavioural and environmental rules.

Term	Behavioural rule	Environmental rule
A	ID of the cell type for which that reaction is available	ID of the cell type for which that reaction is available
B	ID of the cell type resulting from the execution of that rule	Result of the interaction, i.e. amount of nutrients/oxygen consumed, change in stiffness
C	Optional. Additional ID of the cell type resulting from the execution of that rule. Used to model cell doubling and migration	Not present
D	Probability of the reaction. This can be a function of any combination of the variables reported in Table 3, making it possible to simulate complex dynamics and describe realistic cell behaviours	Tag identifying the specific interaction modeled. “environment (Glc)”, “environment (O2)” and “environment (YM)” are currently available. Use the variable “U” to set glucose and oxygen consumption to reference values (glucose uptake: 16.7 10^−12^ g/cell/h^49^, oxygen uptake: 2.93 10^−15^ g/cell/h^50^

**Table 3:** Variables that can be used to define the probability of occurrence of each rule.

Variable	Note
TIME	Current iteration divided by the total simulation length
TD	Iteration at which the current cell die
Glc	Local glucose level divided by its concentration in the media
O2	Local oxygen level divided by its concentration in the incubator
#C	Number of cells of type # divided by the the maximum population cardinalit
AGE	Cell age divided by the current iteratio
TLD	Number of iterations since the last division of the current cell divided by the current iteration
YM	Local Young’s modulus divided by the initial scaffold stiffness

**Figure 8:**
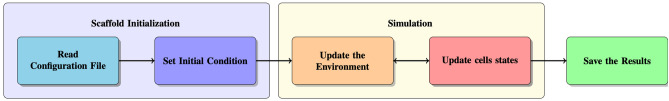
Flowchart describing the main steps of a SALSA simulation.

This information is provided, together with the characteristics of the scaffold (Young’s modulus, the length of its side and resolution), the initial condition (total number of cells and the percentage prevalence of each cell type) and the experimental specifications (length, volume and frequency of media change) in the configuration file that is loaded at the beginning of the simulation (Fig. 8). This organization effectively separates the definition of the characteristics of the system from its simulation, thus improving its generality and usability.

As shown in Fig. 8, each simulation starts with the setting of the initial conditions (e.g. scaffold properties, number of cells and their position). This includes the definition of a scale factor between the experimental and simulated population. Indeed, it is generally not possible to simulate the same number of cells used for an in-vitro experiment. Thus, the volume of the average eukaryotic cell (4.2 10^−6^ mm^3^^51^ together with the initial population cardinality and the scaffold dimensions, are used to compute the fraction of scaffold initially occupied by the cells in a wet-lab experiment of interest. This number is then multiplied by the total capacity (i.e. resolution) of the in-silico matrix to determine the starting number of virtual cells. The ratio between the two populations (in-vitro and in-silico) is then used to adjust the nutrients and oxygen consumption in the environmental rules. Although not entirely accurate, this strategy maintains realistic glucose and oxygen dynamics while optimizing the computational cost. Obviously, if sufficient computational resources are available, increasing scaffold resolution will result in a larger in-silico population and thus in a reduced gap with the corresponding in-vitro assay (see the Supplementary material for a more detailed analysis of the dependence of the computational time on the configuration parameters).

Within each iteration 2nd Fick’s law (Eq. 3) is solved to update the distribution of glucose and oxygen (C). In this work we set the diffusion coefficients (D) of the molecules to those measured in water^52,53^, since it was showed^54^ that the scaffold structure does not influence this process.

3 $$ \frac{\delta C}{{\delta t}} = D\Delta C $$

Culture medium substitution is modelled resetting, in every point of the scaffold, the glucose concentration to the starting value.

The status of every cell in the population is then updated, through the execution of all the environmental rules and one transition rule, chosen according to its probability. This procedure is repeated for each iteration. At the end of the simulation the results are saved in structured text files.

### SALSA parameters and simulation

For this study SALSA was used to simulate the behaviour of two human breast cancer cell lines (MCF-7 and MDA-MB-231) grown in collagen scaffolds during 10 days. The same cell types (dead, quiescent and proliferant) and rules were used to simulate both systems, while the probabilities of specific reactions. i.e. their likelihood of occurrence, were adjusted to account for the differences between the two populations.

In particular, all cell types can either maintain their current status, or a) duplicate, migrate, become quiescent when proliferant, b) proceed toward another state when quiescent, or c) degrade when dead. The coefficients in Table 4, (that multiply the corresponding behavioural rules) were used to differentiate the behaviour of the two cell lines. These values were determined combining experimental results obtained for the specific cell model considered in this work (difference in proliferation, migration and LOX production between MCF7 and MDA-MB-231), evidences from the scientific literature^55,56^ and computational optimization. Overall they favour cell duplication, migration and transition toward the proliferative state in the more aggressive MDA-MB-231 cell line.

**Table 4:** Differences between the probability of occurrence of the behaviour rules for the two considered cell lines (MCF7 and MDA-MB-231).

	MCF 7	MDA-MB-231	References
Duplication–degradation	0.38	0.44	^18,55^
Transition from quiescent to proliferant state	0.005	0.02	–
Migration	0.008	0.01	^56^
LOX expression	0.000003	0.003	^39^

Additionally a differential expression of the Lysyl oxidase enzyme (LOX) is modelled in the two cell lines. This enzyme, that is responsible for collagen cross-linking, was shown in^39^ to be connected with the ability of the cells to remodel their local environment, increasing the stiffness of the surrounding ECM. A more extensive description of the rules used in this work, and of the corresponding probability functions and parameters, is available in the Supplementary Material.

The system presented in^57^ was considered to model the characteristics of the scaffolds. In particular the initial ECM stiffness, specified through its Young’s modulus, was set to 47 kPa and an initial population equivalent to 5 M proliferant cells (see previous section) was simulated during 10 days in DMEM changed every 24 h (time resolution 1 h).

Some simplifications were also added. The cylindrical shape of the scaffold was substituted with a cubic one, that better adapts to the rectangular grid of the model and allows for an easier definition of important features of SALSA (e.g. each cell’s neighbourhood). This choice allows to preserve the generality of the framework, easily adapting to different scaffold types and shapes, while maintaining strong ties with the dimensions of the in-vitro structure (side length 1 cm, resolution 10 layers).

### Global sensitivity analysis

The polynomial chaos expansion method (as implemented in Uncertainpy^58^ was used to conduct a global sensitivity analysis of our model. The reference values for all the parameters were set as detailed in the supplementary material and a variation of 40% (uniformly distributed and centered on the reference value) was considered. This study allowed to determine both how each parameter (Table 1) influences the output variables (cell density and average Young’s modulus) and how stochasticity propagates throughout the model. The former consisted in the determination of the first order and total Sobol indices while the latter produced a representation of how standard deviation varies with the considered parameters throughout the simulation.

Behavioural (a, b, c, d, e) and environmental (U_Glu_, U_O2_, U_YM_, s) parameters were considered separately to contain the memory requirements of the analysis. This choice is expected to have limited effect on the results, due to the negligible difference between total and first order Sobol indices.

### Scaffold preparation

Collagen scaffolds were synthesised and characterised as described in^57^ using chemicals purchased from Sigma Aldrich. Briefly, bovine type I collagen was suspended in 1% acetate buffer (pH 3.5) and adjusted to pH 5.5 with 1 M NaOH. After three washes with deionised water, collagen was cross-linked in an aqueous solution of 2.5 mM 1,4-butanediol diglycidyl ether (BDDGE) for 24 h.

Collagen was again washed with deionised water, casted in Teflon moulds (9 mm diameter) and freeze dried. An optimised freezing and heating ramp was used to obtain the desired pore size and porosity that were characterised through scanning electron microscopy, water and ethanol infiltration methods. Mechanical testing of the scaffolds was performed as previously described in^39^.

### 3D cell culture

The human breast cancer cell lines used in this study were both obtained from the American Type Culture Collection (ATCC) and cultured in DMEM supplemented with 10% fetal bovine serum, 1% penicillin–streptomycin and 1% glutamine (PAA) at 37* oC* in a 5% CO2 atmosphere. Cells were seeded on top of dry sterile scaffolds and left to adhere during 24 h before the beginning of the experiment. Medium was replaced every 24 h.

### Cell density evaluation

The number of cells that populated the scaffolds was assessed over time quantifying the total amount of DNA using the PicoGreen dsDNA assay (Invitrogen). Briefly, DNA was extracted from the scaffolds using the DNeasy Blood and Tissue Kit (Qiagen) and then 100 μL of DNA mixture were added to 100 μL of PicoGreen reagent working solution. Fluorescence was measured with a microplate reader (FLUOstar OPTIMA BMG LABTECH) with excitation and emission wavelengths at 480 and 520 nm, respectively. The conversion factor of 7.7 pg DNA/cell was used to determine the total number of cells. A cytometry analysis allowed the quantification of the percentage of viable cells. Cells were harvested from the scaffold by enzymatic digestion with Collagenase type I (Merck Millipore), and stained with 50 μM calcein AM and 2 mM ethidium homodimer-1 (Invitrogen). The cell suspension was assayed using BD FACSCanto (Beckmann Coulter).

Finally, cell density was calculated as the fraction of living cells within the scaffold normalised with respect to the cardinality of the initial population. The same calculation was applied for the in-silico data, considering the sum of proliferating and quiescent cells as viable cells.

### Confocal microscopy

Confocal images of the cells were acquired as in^40^, using an A1 laser confocal microscope (Nikon Corporation, Tokyo, Japan) and the NIS Elements software (Nikon Corporation, Tokyo, Japan). Samples were fixed with 4% paraformaldehyde at room temperature and stained with 10 μ M/ml DRAQ5 (ImmunoChemistry Technology, Bloomington, MN, USA).

### Statistical analysis

The concordance between in-silico simulations and in-vitro data was measured computing the mean average percentage error (MAPE) at each available time point (1, 3, 7, 10 days) for each simulation independently. This approach was applied to both the cell density and the stiffness data.

Results are reported as mean and 95% confidence intervals, unless stated otherwise, since most of the presented data are not normally distributed (Shapiro–Wilk test, *p* = 0.05). As such, when independence between two conditions was evaluated, the Kruskal–Wallis test was used.

## Supplementary information

Supplementary Information 1.

Supplementary Information 2.
