# Supplementary material for: An in-silico study of cancer cell survival and spatial distribution within a 3D microenvironment
Source: Sci Rep. 2020 Jul 31;10:12976. doi: 10.1038/s41598-020-69862-7 (PMC7395763; doi:10.1038/s41598-020-69862-7)
Supplement: Supplementary file 1 — Supplementary Information 1. [file 41598_2020_69862_MOESM1_ESM.pdf]

# An *in-silico* study of cancer cell survival and spatial distribution within a 3D microenvironment

## Supplementary figures and tables.

**Marilisa Cortesi[1,*], Chiara Liverani[2], Laura Mercatali[2], Toni Ibrahim[2], and Emanuele Giordano[1,3,4]**

[1] University of Bologna, Department of Electrical, Electronic and Information Engineering "G. Marconi", Cesena (FC) Italy.

[2] Istituto Scientifico Romagnolo per lo Studio e la Cura dei Tumori (IRST) IRCCS, Osteoncology and Rare Tumors Center, Meldola (FC) Italy.

[3] University of Bologna, Advanced Research Center on Electronic Systems (ARCES), Bologna (BO) Italy.

[4] University of Bologna, BioEngLab, Health Science and Technology, Interdepartmental Center for Industrial Research (HST-CIRI), Ozzano Emilia (BO) Italy.

[*] marilisa.cortesi2@unibo.it

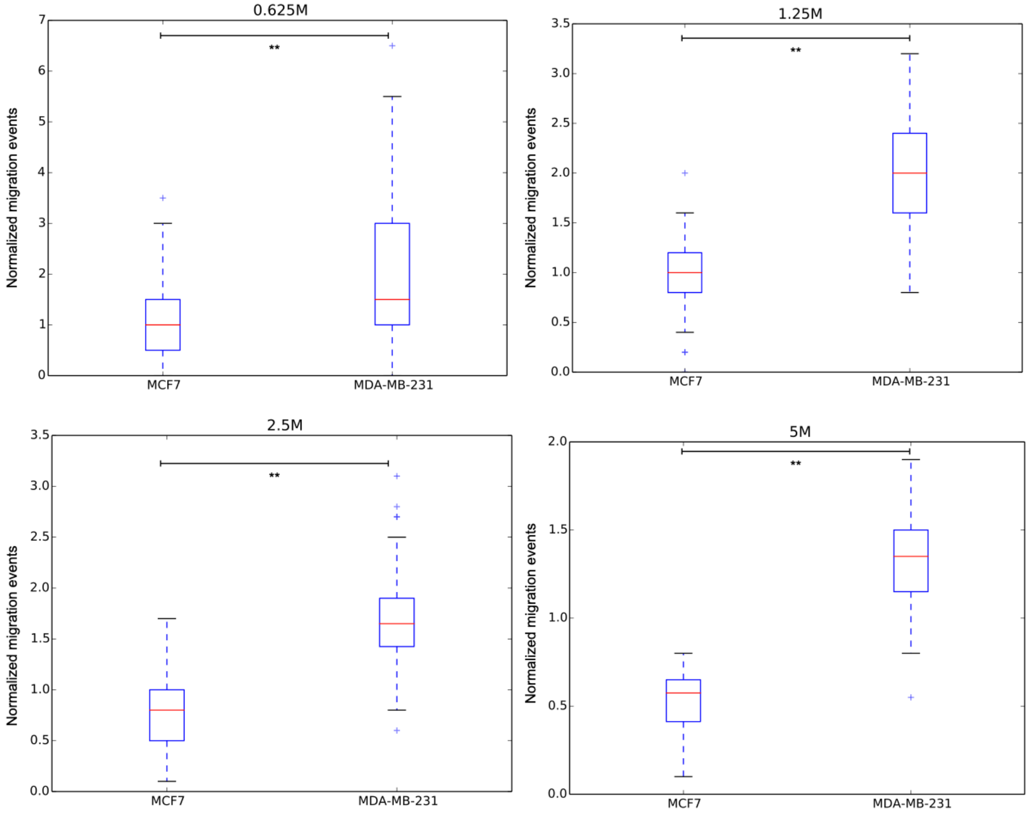


**Figure 1**: Distribution of the number of migratory events normalized by the initial cell density. For all cardinalities MDA-MB-231 cells migrate about twice as much when compared with MCF7 cells.

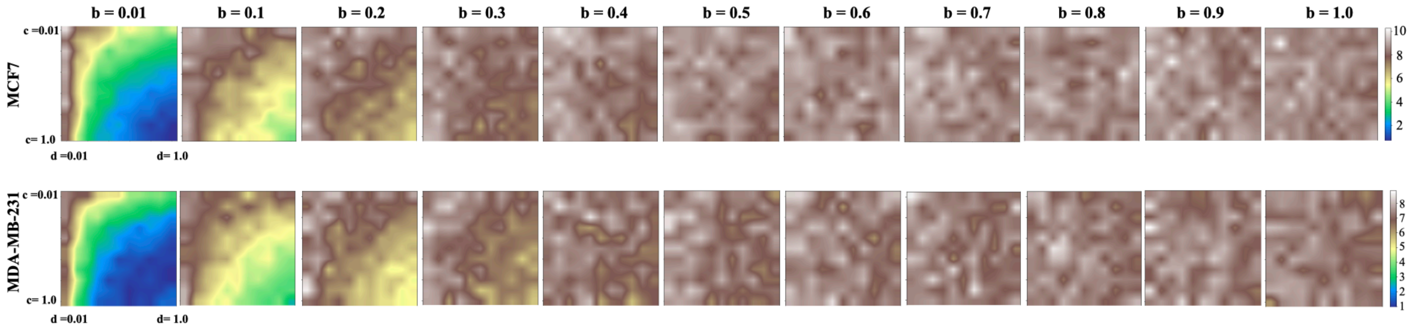


**Figure 2**: Errors corresponding to the wide range parameters sets. A bilinear interpolation has been applied to smooth the transitions between different values.

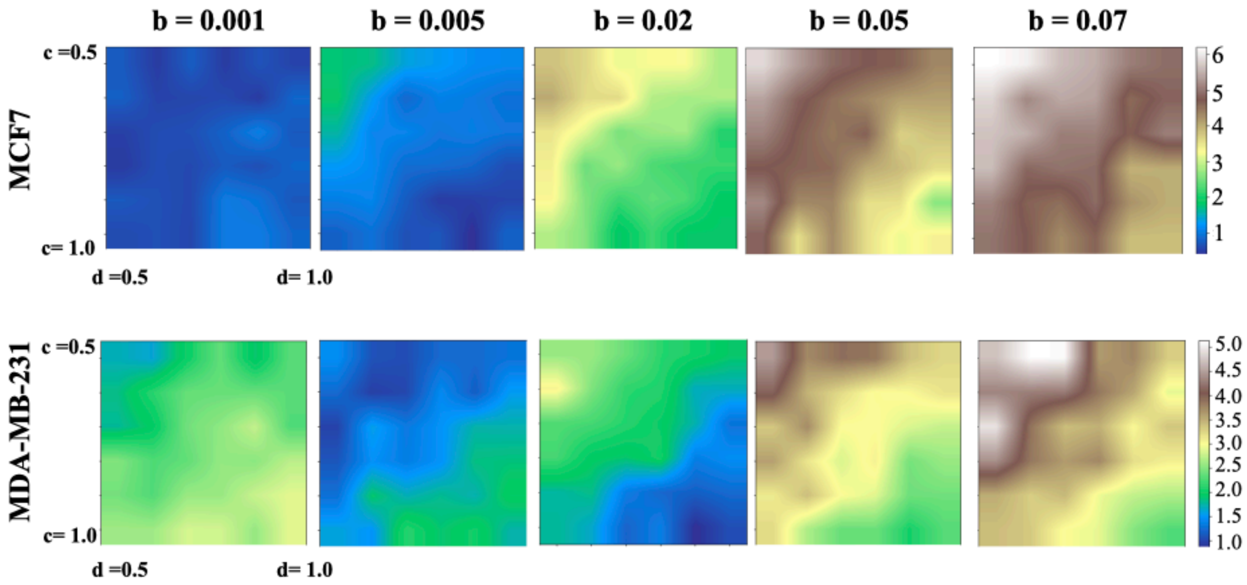


**Figure 3:** Errors corresponding to the narrow range parameters sets. A bilinear interpolation has been applied to smooth the transitions between different values.

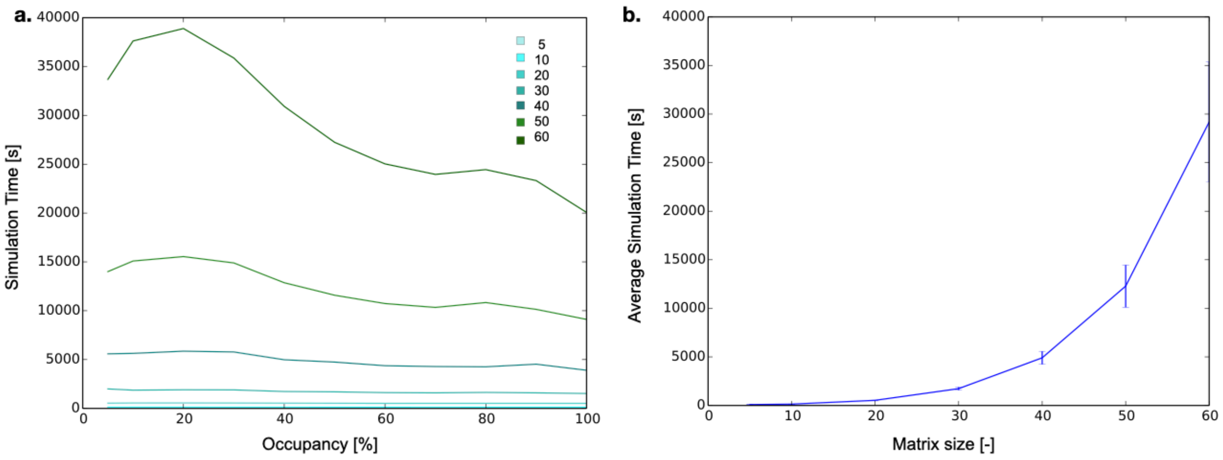


**Figure 4:** Computational time. In a. the simulation time associated with each combination of scaffold size and occupancy is reported, while in b. the average simulation time for each matrix size is shown (data reported as mean and standard deviation).

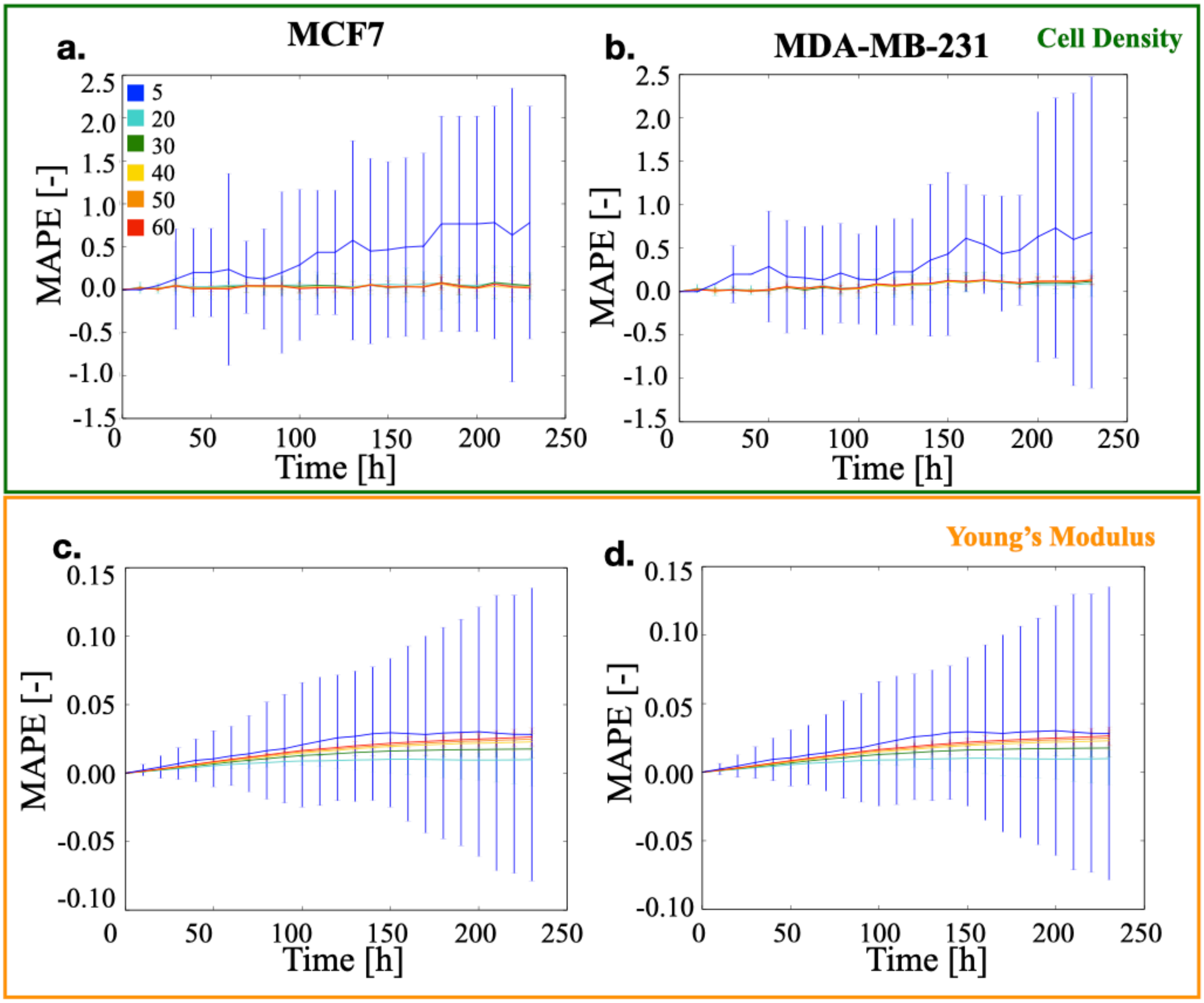


**Figure 5:** Analysis of the influence of the lattice size on the simulation's outputs. For graphical clarity only one in ten points is reported.

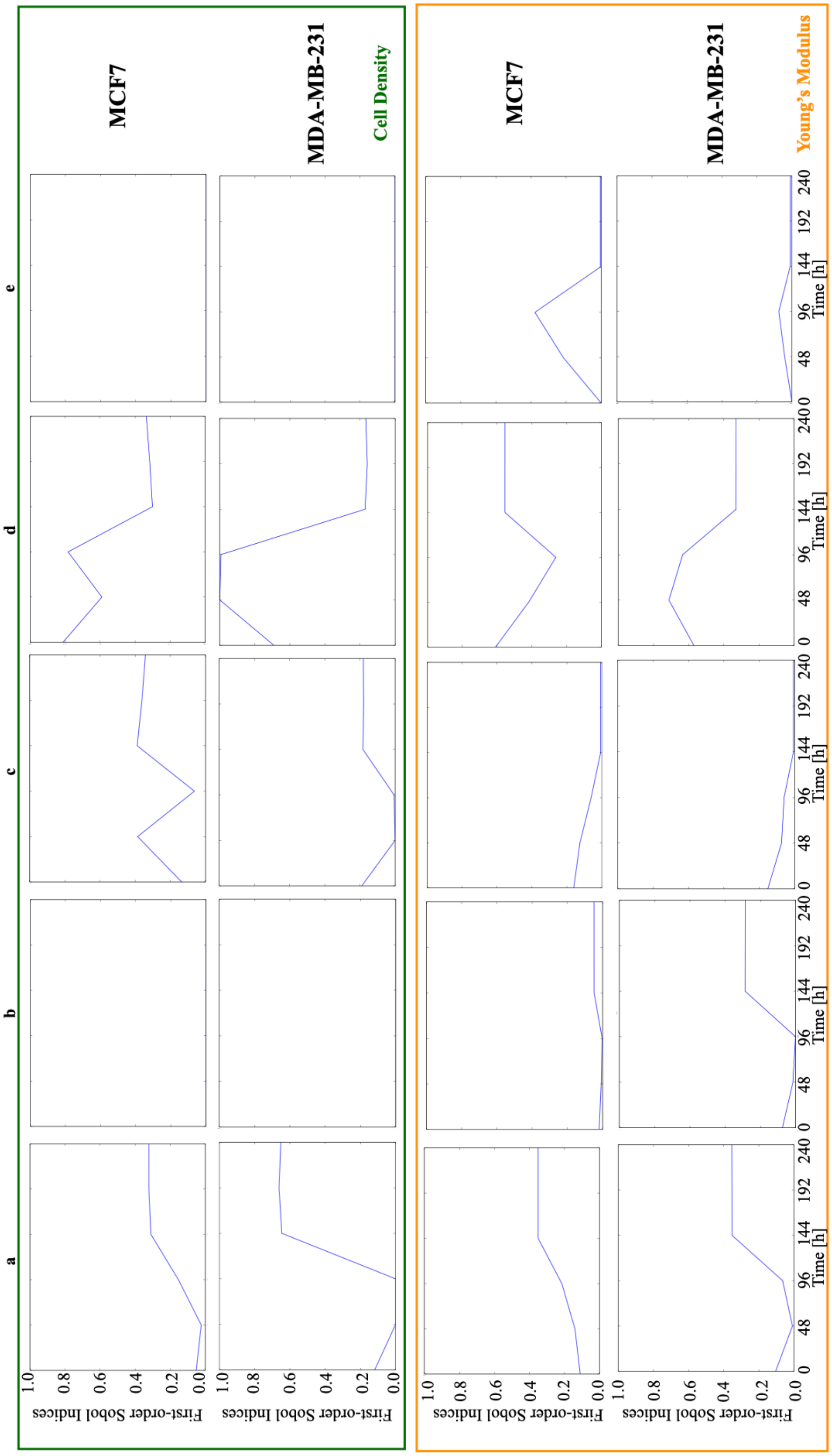


**Figure 6:** First order Sobol indices computed for each behavioural parameter and output. Both the x and the y axis are shared among all panels.

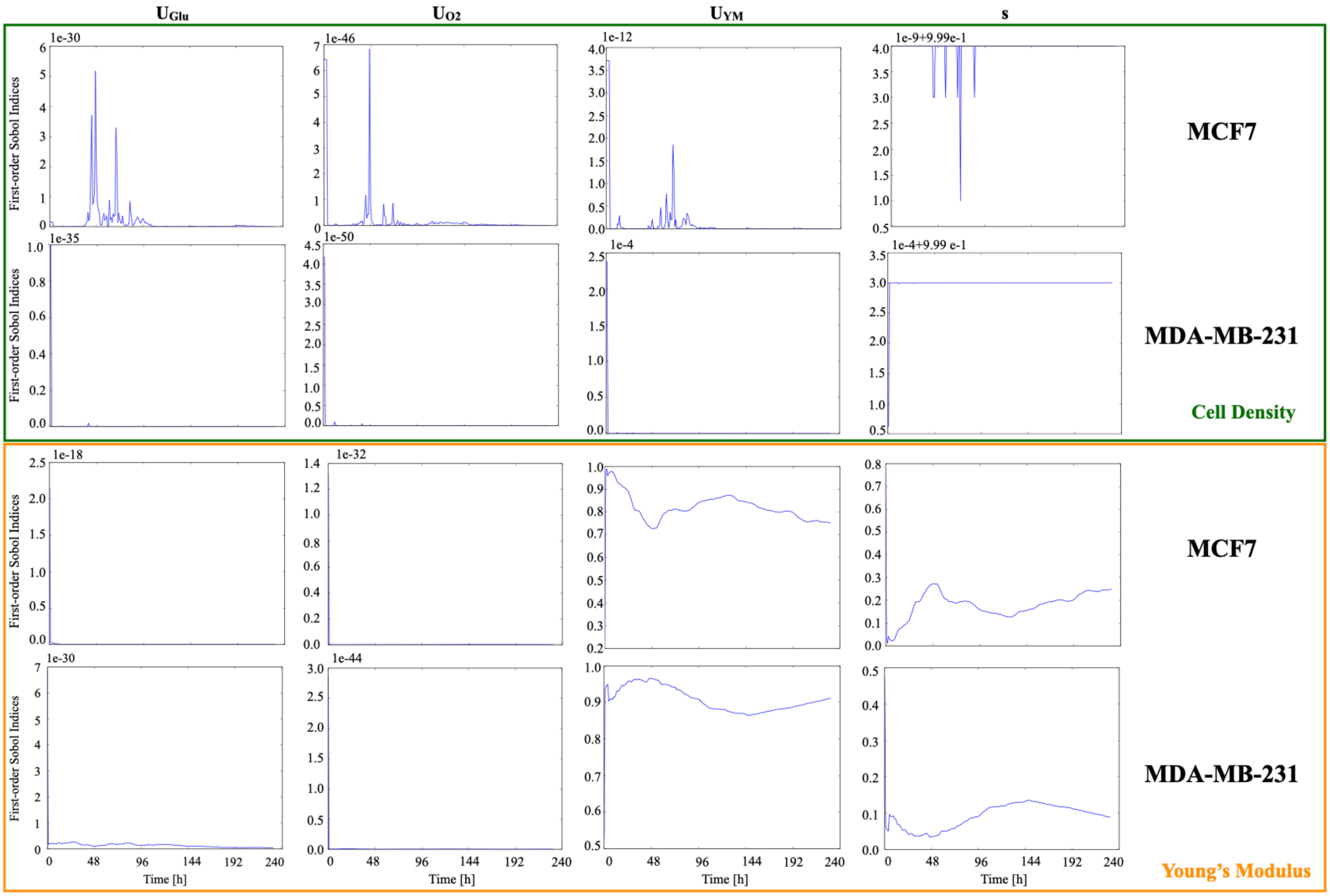


**Figure 7:** First order Sobol indices computed for each environmental parameter and output. The x axis is shared among all panels.

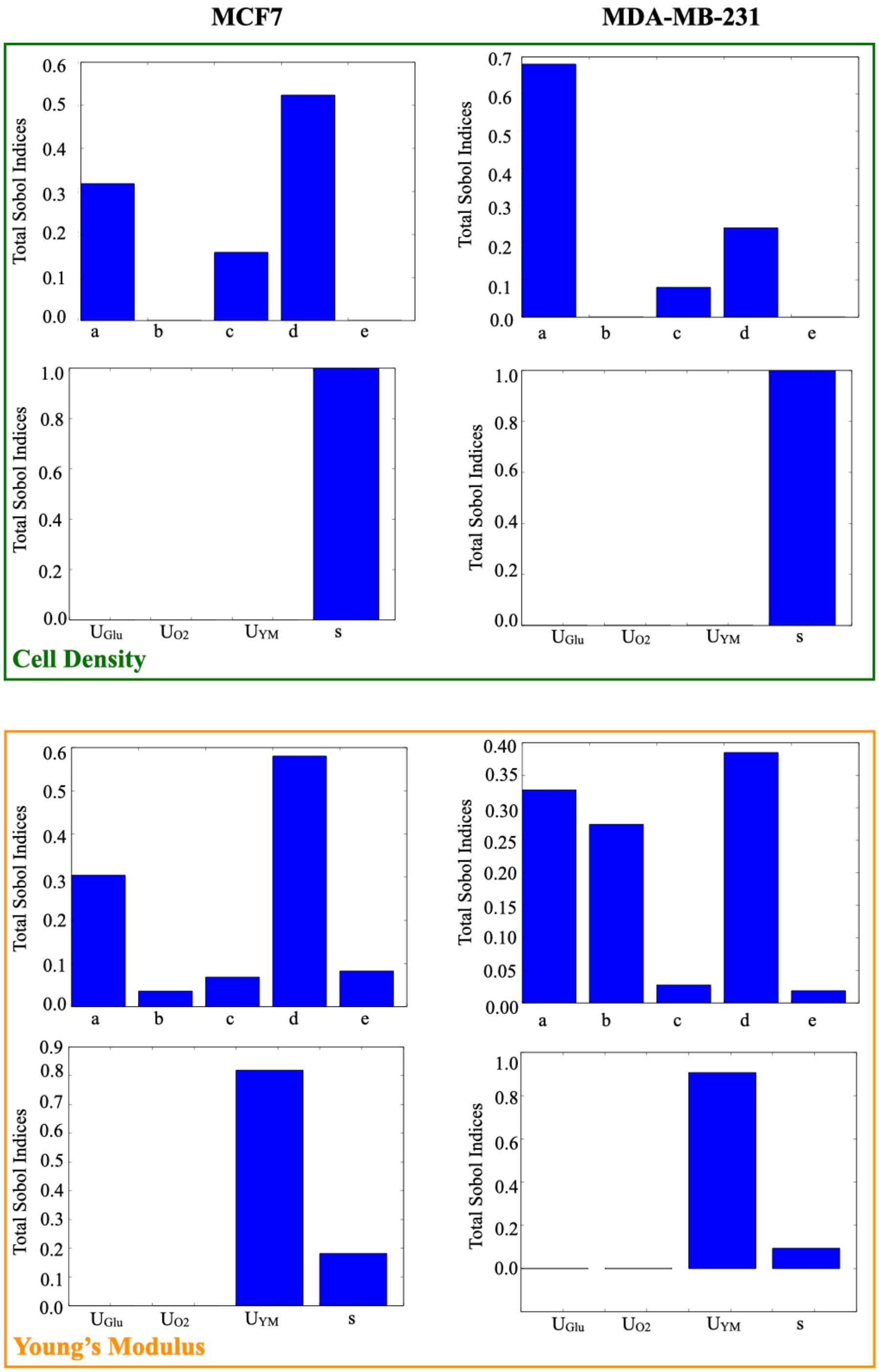


**Figure 8:** Total average Sobol indices computed for each environmental parameter and output.

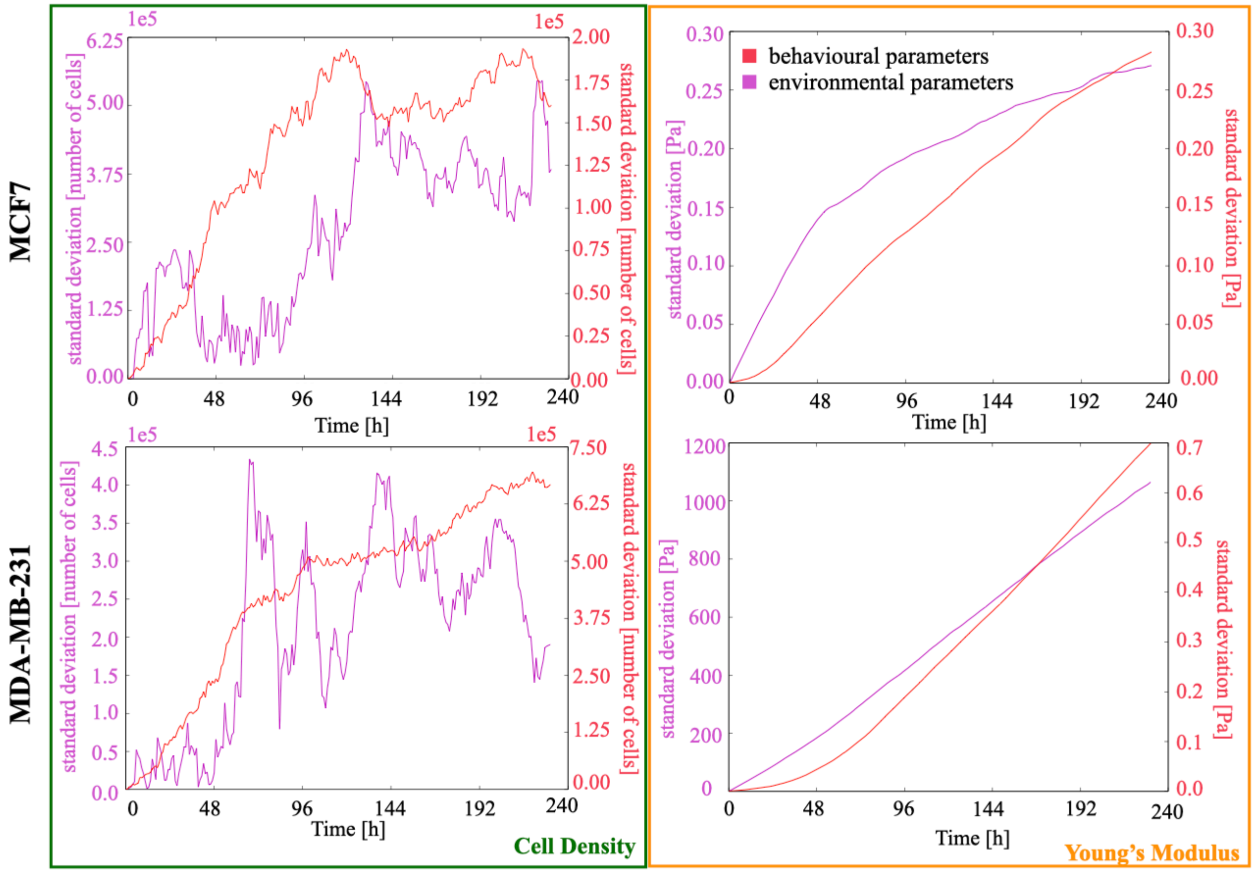


**Figure 9:** Standard deviation due to the variation of the parameters involved in the sensitivity analysis.

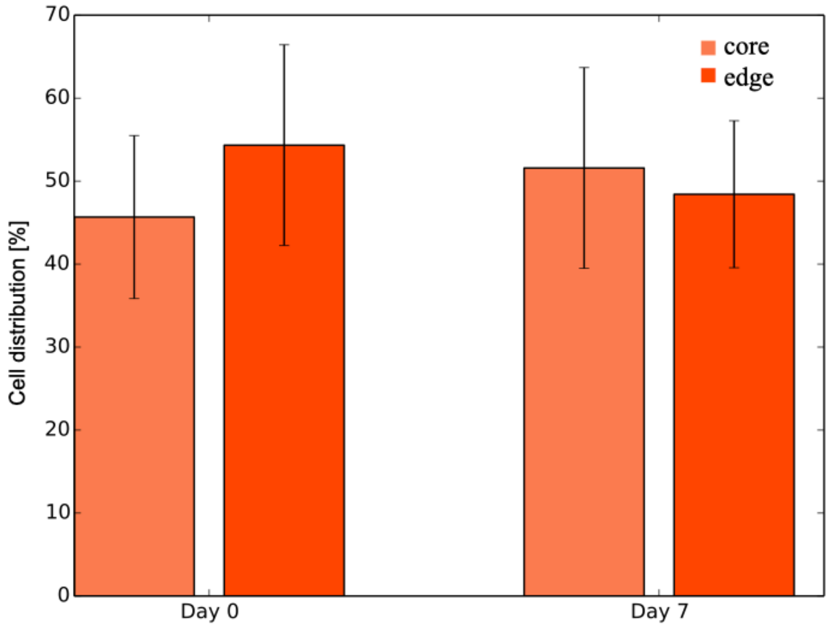


**Figure 10:** Experimental cell distribution within the scaffold. A starting population of 1M cells reduced importantly the difference in cell density between core and edge regions measured in [8]. Indeed at the end of the experiment the cell population is approximately uniformly distributed within the whole structure.

| Coefficient | MCF 7 | MDA-MB-231 |
|---|---|---|
| a | 0.38 | 0.44 |
| b | 0.005 | 0.02 |
| c | 1 | 1 |
| d | 0.9 | 0.9 |
| e | 0.008 | 0.01 |
| f | 0.000003 | 0.003 |

**Table 1:** Values of the coefficients used for the simulation of MCF7 and MDA-MB-231 cells

| Parameter | value |
|---|---|
| CPU | 32 Intel(R) Xeon(R) CPU E5-2640 v2 @ 2.00GHz |
| RAM | 65GB |

**Table 2:** Specifications of the workstation used for the SALSA simulations

| Parameter | Value |
|---|---|
| Side | 1 cm |
| Layers | 10 |
| Initial  population composition | all proliferant cells |
| Initial population cardinality | 625 K, 1.25 M, 2.5 M, 5M |
| Iteration | 240 |
| Media replace frequency | 1 day |

**Table 3:** Parameters of the virtual scaffold. The complete configuration files are available, together with SALSA source code, at http://www.mcbeng.it/en/category/software.html
