# Supplementary material for: An in-silico study of cancer cell survival and spatial distribution within a 3D microenvironment
Source: Sci Rep. 2020 Jul 31;10:12976. doi: 10.1038/s41598-020-69862-7 (PMC7395763; doi:10.1038/s41598-020-69862-7)
Supplement: Supplementary file 2 — Supplementary Information 2. [file 41598_2020_69862_MOESM2_ESM.docx]

Supplementary Material:

An *in-silico* study of cancer cell survival and spatial distribution within a 3D microenvironment

Marilisa Cortesi, Chiara Liverani, Laura Mercatali, Toni Ibrahim, Emanuele Giordano

**Configuration file and simulation**

SALSA was developed so as to maintain a strict separation between the definition of the model and the simulator. The latter was implemented for this work using python (v3.6) and is freely available at http://www.mcbeng.it/en/category/software.html.

The former, on the other hand, relies on a configuration file with a clearly defined structure (reported below).

MODEL:
Cell Types:
cell Line = MDA-MB-231 1 = Dead
2 = Quiescent
3 = Replication

Rules:

0 = 1-> 0, a* (TIME-TD)
1 = 1-> 1, 1-(a* (TIME-TD))
2 = 1-> 0, environment (Glc)
3 = 1-> 0, environment (O2)
4 = 2-> 3, b*(Glc+O2)/(1C+2C+3C+AGE)
5 = 2-> 1, c*(1C+2C+3C)/(Glc+O2)
6 = 2-> 2, 1-(b*(Glc+O2)/(1C+2C+3C+AGE))-(c*(1C+2C+3C)/(Glc+O2))

7 = 2-> 0.5*U, environment (Glc)
8 = 2-> 0.5*U, environment (O2)
9 = 3-> 2, d*(AGE+1C+2C+3C)/(Glc+O2)
10 = 3-> 3+3, a*TLD/(YM+Glc+O2)
11 = 3-> 0+3, e*O2/(YM+1C+2C+3C)
12 = 3-> 3, 1-(d*(AGE+1C+2C+3C)/(Glc+O2))-(a*TLD/(YM+Glc+O2))+ -(e*O2/(YM+1C+2C+3C))
13 = 3-> U, environment (Glc)
14 = 3-> U, environment (O2)
15 = 3-> f, environment (YM)

Scaffold:

material = collagen

side [cm] = 1

layers = 10

porosity [%] = 87

INITIAL CONDITIONS:

Cell Types:
1=0
2=0

3 = 100

total=5M

Scaffold:

seeding = Random
iterations = 240
media = RPMI
media replace frequency [day]= 1

volume per scaffold [ml] = 4

flow rate [ml/h] = 0

It comprises two main sections: “Model” and “Initial Condition”. The former is used to define the characteristics of the system (structural properties of the scaffold, cell types and their behaviour) while the latter specifies the initial state and the experimental conditions.

Three subsections can be identified within the model section. The first one, “Cell Types” identifies the cell line and defines each cell type, through the association of each label to a unique ID. In the present study we considered proliferant, quiescent and dead cell types, however this formulation allows the user to define as many cell types as needed.

Cell behaviour is instead specified through transition rules (“Rules” subsection). A detailed description of their features and parameters is reported in the next section.

The final subsection of the “Model”, called “Scaffold” details the characteristics of the matrix (material, side length, number of layers and porosity). These values are used to define the cubic lattice used for the simulation, a 3D matrix programmable with a desired number of layers, the spatial resolution (side [cm]/layers) and the material’s Young’s modulus (using material and percentage porosity to solve equation 1 using the parameters [E0, b, c] in the materials folder of the software).

$E=E_{0}\cdot e^{-b\cdot p+c}$ (1)

The “Initial Condition” section defines the characteristics of the starting population (total number and percentage prevalence of each type) and of the experiment (“Scaffold section”). In the latter the type of seeding (at present only Random is implemented), the number of iterations, the type of media used, its replacement frequency and the volume used for each scaffold are required. These data are used to set the length of the simulation, initialize the glucose distribution and simulate media change. The flow rate field will allow for the simulation of perfusion flow throughout the scaffold. At present it must be set to 0 ml/h as this feature has not yet been implemented.

Each SALSA simulation proceeds as shown in Figure 8 of the main document. At the beginning, the information in the configuration file is read and interpreted, this allows for the initialization of the main variables of the simulation, four 3D cubic lattices containing:

- the position of the cells and their type (as specified by the “Cell Types” subsection of the “Initial Configuration”.
- the distribution of glucose within the scaffold (uniform and coherent with the amount of glucose in the volume and type of media specified by the user).
- the distribution of oxygen within the scaffold (uniform and coherent with standard cell culturing conditions - air with 5% CO2).
- the distribution of Young’s Modulus (uniform and obtained as detailed above. Alternatively, the numerical value of the Young’s modulus can be substituted to the material and the porosity set to 0).

Once this and other service variables (e.g. a lattice keeping track of each cell’s age) have been initialized, the simulation starts. Throughout this step the environment and the cells states are alternatively updated for the number of times specified in the configuration file (iterations). This involve the simulation of the diffusion of glucose and oxygen within the considered time step using the 2nd Fick’s law and the execution of one behavioural rule, chosen according to its probability, and all the environmental rules. To this end, the probability of each behavioural rule is computed, solving the equations defined in the configuration file using the current value of the considered variables, and their value is normalised so that the sum of all the probabilities is equal to one. A random number is then generated and used to determine which rule should be executed.

When an environment rule is identified, the amount of nutrients/oxygen
or the change in stiffness specified in the configuration file are added to/subtracted from the corresponding variable. This simulates the consumption of resources
by living cells and the activity of the Lysyl oxidase enzyme (LOX) that was shown in [1] to provide MDA-MB-231 cells with the ability to remodel collagen and increase its density, hence its stiffness. Within this step, the consumption of resources affects only the specific voxel occupied by the cell, while the stiffness increase also affects its neighbourhood.

After the updating of all the cells in the population, the main variables of the simulation (type and position of every cell, nutrients and stiffness distributions), associated with the corresponding time point, are saved as output variables which are printed in formatted text files that allow to reconstruct every step of the analysis at the end of the simulation.

**SALSA models of MDA-MB-231 and MCF7 cells**

The behaviour of the two breast cancer cell lines considered in this study was formalized through the set of rules reported in the configuration file above.

Rules 0 to 3 refer to the dead cells (type 1) and describe both their behaviour and resources consumption. In this case no glucose and oxygen are consumed (rules 2 and 3) and the only possible transition is toward state 0 (rule 0). As this represents an empty voxel, the only action available to dead cells is degradation, beside remaining in their state (rule 1). The probability of degrading was considered to be proportional to the time elapsed from cell death (TD) and the coefficient “a” was set so as to reflect the differential duplication rate of the two considered cell lines (Supplementary Table 1 and next section).

Quiescent cells (type 2) can transition toward both the proliferant and the dead state (rules 4 and 5 respectively), beside maintaining their status (rule 6). Additionally they consume oxygen with a rate that is half of that experimentally measured [2, 3] to reflect the reduced metabolism of these cells [4]. The probability of transitioning to a proliferative state was formalized so as to be directly proportional to the local amount of resources (glucose and oxygen) and inversely proportional to the total population (1C+2C+3C) and the cell’s age. As such the proliferative status is more likely to be achieved if the local conditions are favourable, the cell is young and the scaffold not too densely populated. On the contrary a high cellular density and low oxygen and glucose level are associated with cell death (rule 5). In this case there is no dependency on cell age as quiescent cells can survive for a long time within the organism [4] and, in this model, they cannot replicate. As for dead cells the values of the coefficients in rules 4 and 5 are reported in Supplementary Table 1 and the procedure used to define them is described in the next section.

Proliferant cells (type 3) exhibit a more complex behaviour that comprises transition to quiescency (rule 9), duplication (rule 10), migration (rule 11), resources consumption (rules 13 and 14) and extracellular matrix (ECM) stiffening (rule 15), beside maintenance of the current status (rule 12). The probability of rule 9 is the reciprocal of that defined for rule 4, as we considered that the same factors that promote the transition toward proliferation could also inhibit it. Duplication was modelled so as to be directly correlated with the time elapsed from that cell’s last division (TLD). This choice allows for an approximately constant duplication rate without imposing specific time points for this process. At the same time, scarce resources and stiff local ECM reduce the likelihood of cell doubling. This is akin to what happens in-vitro as population growth is reduced when glucose and oxygen are low and a rigid substrates can limit cell movement [5]. The probability of migration was formalized so as to be proportional to the local concentration of oxygen, to provide cells with a strategy to escape the hypoxic core that has been shown to often form in-vivo at the core of tumoral masses [6]. This formalization disregards the dependency of the probability of migration on the concentration of glucose [7]. This choice allowed us to better highlight the role of oxygen in migration (see also [1, 8]). The addition of Glc to the numerator is likely to further promote migration toward the edge. Additionally a stiff ECM and a high cellular density decrease the probability of migrating. Indeed a high cell density curbs cell movement [9] and so does high scaffold density. In this work we considered this latter variable to be the only determinant of the increase in stiffness measured in scaffolds where MDA-MB-231 cells were cultured. This simplification does not take into account the ability of invasive cells like MDA-MB-231 of remodelling the ECM to create preferential routes for migration [8]. This more ordinate structure favours migration, rather than hindering it, and is associated with a marked increase in stiffness. Its large scale organisation, however, requires external mechanical cues (stress or strain) to allow for fibers alignment [10]. As no mechanical stimuli were applied, we decided to disregard this phenomenon and maintain a more generally applicable proportionality between matrix density and stiffness. This simplification does not change the expected behaviour. Indeed the number of migratory events, normalized by the cardinality of the initial population, was about twice as much for MDA-MB-231 when compared with MCF7 cells (Figure 1).

As for the environment rules, proliferating cells consume resources at experimentally measured rates [2, 3] and increase the stiffness of their environment proportionally to their expression of Lysil-oxydase (LOX), as described in [1] . Again the values of the coefficients considered for these rules (a, d, e and f) are reported in Supplementary Table 1 and the explanation of their derivation is detailed in the next section.
When executing a rule with two outputs (e.g. migration, proliferation) the additional voxel involved is chosen as the one in the neighbourhood with the highest concentration of glucose and oxygen (sum of their local concentrations both normalized with respect to their reference values). If more than one option is available, the proliferation/migration endpoint is chosen randomly.

**Parameters identification**

The identification of the coefficients used to modulate the probability rules described in the previous section integrated information from the scientific literature, experimental data acquired on the specific model considered and numerical optimization. This approach was necessary as some data were not available for the considered system or not easily measurable in-vitro. Specific care, however, was taken when selecting these values: data acquired in 2D or for other cell lines were not considered.

Coefficient “a” was identified combining the doubling rate of MDA-MB-231 cells measured in [11] in 0.5% collagen scaffolds, the density of the 3D support used in this study (1 %) and its Young’s modulus (47 KPa) to solve the equation:

$$a\cdot\frac{DT+0.5\cdot DT}{YM}=1$$

where DT is the doubling time and YM the Young’s modulus. In this case the DT measured in [11] was increased by half, to ensure a doubling probability of 1 in the considered time-frame, and the Young’s modulus was halved to account for the different scaffold density.
The “a” coefficient for the MCF7 cells was obtained reducing that of the MDA-MB-231 by 15 % as this is the difference at 24 h between the proliferative rates of the two considered cell lines (see Figure 1 of the main text). These same values were used also for the degradation rule as in [12] the half life of most intracellular proteins is determined to be approximately equal to the doubling rate.

$E=\Sigma_{i}|vitro(i)-silico(i)|$ (2)

Coefficients “b”, “c” and “d” were estimated through numerical optimization, as the probabilities of transitioning between different cell states are not easily measurable. The range of possible values was screened considering all possible parameter sets obtained combining the following values (0.1, 0.2, 0.3, 0.4, 0.5, 0.6, 0.7, 0.8, 0.9, 1). In all cases the error function in Equation 2 (where the index “i” associated simulated and experimental results corresponding to the same timepoint) was used to quantify the goodness of each parameter set, considering the average cell density of 5 simulations. The results of this analysis are reported in Supplementary Figure 2 where different surfaces correspond to alternative values of “b”. “c” and “d” vary over the y and x axis respectively and the color codes for the error. The area associated with the best approximation of the experimental data corresponds to low values of “b” and high values of “c” and “d”. As such a more refined analysis of this region was conducted, applying the same procedure and considering all the possible combinations of the following values:

b= 0.001, 0.005, 0.01, 0.02, 0.05, 0.07

c, d= 0.5, 0.6, 0.7, 0.8, 0.9, 1

The errors computed in this second optimization process are reported in Supplementary Figure 3 and allowed to identify the values of “b”, “c” and “d” for the two considered cell models.

The coefficient identified with the letter “e” was retrieved from a combination of the data reported in [13] and knowledge about the considered experimental model. In particular the migration speed of MDA-MB-231 cells was determined to be 10 μm/h that, considering the size and resolution of the virtual scaffold, corresponds to 0.01 layers/iteration. A previous characterization of our experimental model (data not shown) determined a migration rate for MCF7 cells 20% lower than the more invasive MDA-MB- 231 cell line.

Finally the rate of modification of the scaffold Young’s modulus (coefficient “f”) was defined empirically so as to obtain, for the MDA-MB-231 cells, an average matrix stiffness at the end of the simulation comparable to that measured at day 10. The ratio between the expression of LOX in the two cell lines [1] was then used to identify this coefficient for MCF7 cells.

**Variables update**

The variables update procedure defined in SALSA allows for the integrated update of discrete and continuous variables.
Initially the nutrients concentration is updated. This includes diffusion of glucose and oxygen and, at the user-specified time points, media change. Successively the status of each cell is updated sequentially. This step entails cycling over all the available rules for that cell type, computing the probability of each behaviour rule and applying all the environmental rules. Finally the behavioural rule to execute is chosen, according to the probabilities of occurrence computed previously, and the status of the cell is updated. The successive iteration is then started, after saving all the relevant variables.

**Computational Time and Spatial Resolution**

The time required for running a SALSA simulation depends on a number of different factors, like the resolution of the domain and the size of the population. To characterize the dependency of the computational time on these parameters we performed a set of simulations in which the resolution of the scaffold and the initial population cardinality were varied over wide ranges:

- Resolution [-]: 5, 10, 20, 30, 40, 50, 60
- Occupancy [%]: 5, 10, 20, 30, 40, 50, 60, 70, 80, 90, 100

To eliminate the effect of changing cell number on the simulation time, the number of simulated cells was maintained constant. Indeed after computing the probability of all the applicable rules, the one maintaining the same proliferant status was forcibly chosen. As shown in Figure 9 a. the scaffold resolution is the most relevant parameter, determining an exponential growth of the simulation time (Figure 9 b.).

The matrix percentage occupancy, on the other hand, acquires significance only when large matrices are considered (more than 50 layers). In this case, large populations tend to be associated with shorter simulations. This suggests that the most computationally intensive part of the simulation is likely the solution of the 2nd Fick’s law, that will produce less uniform distributions when fewer cells are considered. As such, substituting the custom solver with a more optimized one could drastically reduce the computational time. Tables 2 and 3 report the characteristics of the workstation and the parameter configuration used for all the simulations presented in this paper.

Additionally, we decided to study how the size of the lattice influences the results. To this end, five simulations for each lattice size considered in the computational time analysis (5, 20, 30, 40, 50, 60) were conducted, and the simulated cellular density and Young’s modulus were compared to the *in-silico* results presented in the main text through the MAPE metric (Equation 1 of the main text). The results are shown in Figure 5.

With the exception of the lowest resolution (side of size 5 voxels), all tested conditions are associated with low error (below 5%) thus supporting our choice of a lattice of size 10 voxels as the best tradeoff between accuracy and computational cost.

**References**

[1]  C. Liverani, L. Mercatali, L. Cristofolini, E. Giordano, S. Minardi, G. Della Porta, A. De Vita, G. Miserocchi, C. Spadazzi, E. Tasciotti, D. Amadori, and T. Ibrahim, “Investigating the mechanobiology of cancer cell-ecm interaction through collagen-based 3d scaffolds,” Cellular and Molecular Bioengineering, vol. 10, pp. 223–234, 2017.

[2]  K. Birsoy, R. Possemato, F. Lorbeer, E. Bayraktar, P. Thiru, B. Yucel, T. Wang, W. Chen, C. Clish, and D. Sabatini, “Metabolic determinants of cancer cell sensitivity to glucose limitation and biguanides,” Nature, vol. 508, pp. 108–12, 2014.

[3]  I. Streeter and U. Cheema, “Oxygen consumption rate of cells in 3d culture: The use of experiment and simulation to measure kinetic parameters and optimise culture conditions,” Analyst, vol. 136, pp. 4013–4019, 2011.

[4]  J. Valcourt, J. Lemons, E. Haley, M. Kojima, O. Demuren, and H. Coller, “Staying alive, metabolic adaptation to quiescence,” Cell Cycle, vol. 11, pp. 1680–1696, 2012.

[5]  S. Owen and M. Shoichet, “Design of three-dimensional biomimetic scaffolds,” Journal of Biomedical Materials Research, vol. 96A, pp. 1321– 1331, 2010.

[6]  E. Hammond, M. Asselin, D. Forster, J. O’Connor, J. Senra, and K. Williams, “The meaning, measurement and modification of hypoxia in the laboratory and the clinic,” Clinical Oncology, vol. 26, pp. 277–288, 2014.

[7]  L. Liu, G. Duclos, B. Sun, J. Lee, A. Wu, Y. Kam, E. D. Sontag, H. A. Stone, S. J. C, R. A. Gatenby, and R. H. Austin, “Minimization of thermodynamic costs in cancer cell invasion,” PNAS, vol. 110(5), pp. 1686–1691, 2013.

[8]  C. Liverani, A. De Vita, M. S, Y. Kang, L. Mercatali, D. Amadori, F. Bongiovanni, A ansd La Manna, T. Ibrahim, and E. Tasciotti, “A biomimetic 3d model of hypoxia-driven cancer progression,” Scientific Reports, vol. 9, 2019.

[9]  S. Tlili, E. Gauquelin, B. Li, O. Cardoso, B. Ladoux, H. Delano’e-Ayari, and F. Graner, “Collective cell migration without proliferation: density determines cell velocity and wave velocity,” Royal Society Open Science, vol. 5, p. 172421, 2018.

[10]  C. Storm, J. J. Pastore, F. C. MacKintosh, T. C. Lubensky, and P. A. Janmey, “Nonlinear elasticity in biological gels,” Nature, vol. 435, 2005.

[11]  B. J. Kim, S. Zhao, R. Bunaciu, A. Yen, and M. Wu, “A 3d in situ cell counter reveals that breast tumor cell (mda-mb-231) proliferation rate is reduced by the collagen matrix density,” Biotechnology Progress, vol. 31, pp. 990–996, 2015.

[12]  M. Larance, Y. Ahmad, K. Kirkwood, T. Ly, and A. Lamond, “Global subcellular characterization of protein degradation using quantitative proteomics,” Molecular and Cellular Proteomics, vol. 12, pp. 638–650, 2013.

[13]  U. Haessler, J. Teo, D. Foretay, P. Renaud, and M. Swartz, “Migration dynamics of breast cancer cells in a tunable 3d interstitial flow chamber,” Integrative Biology, vol. 4, pp. 401–409, 2012.

**Figure and Table legends**

**Figure 1**: Distribution of the number of migratory events normalized by the initial cell density. For all cardinalities MDA-MB-231 cells migrate about twice as much when compared with MCF7 cells.

**Figure 2**: Errors corresponding to the wide range parameters sets. A bilinear interpolation has been applied to smooth the transitions between different values.

**Figure 3:** Errors corresponding to the narrow range parameters sets. A bilinear interpolation has been applied to smooth the transitions between different values.

**Figure 4:** Computational time. In a. the simulation time associated with each combination of scaffold size and occupancy is reported, while in b. the average simulation time for each matrix size is shown (data reported as mean and standard deviation).

**Figure 5:** Analysis of the influence of the lattice size on the simulation’s outputs. For graphical clarity only one in ten points is reported.

**Figure 6:** First order Sobol indices computed for each behavioural parameter and output. Both the x and the y axis are shared among all panels.

**Figure 7:** First order Sobol indices computed for each environmental parameter and output. The x axis is shared among all panels.

**Figure 8:** Total average Sobol indices computed for each environmental parameter and output.

**Figure 9:** Standard deviation due to the variation of the parameters involved in the sensitivity analysis.

**Figure 10:** Experimental cell distribution within the scaffold. A starting population of 1M cells reduced importantly the difference in cell density between core and edge regions measured in [8]. Indeed at the end of the experiment the cell population is approximately uniformly distributed within the whole structure.

**Table 1:** Values of the coefficients used for the simulation of MCF7 and MDA-MB-231 cells

**Table 2:** Specifications of the workstation used for the SALSA simulations

**Table 3:** Parameters of the virtual scaffold. The complete configuration files are available, together with SALSA source code, at http://www.mcbeng.it/en/category/software.html
